# Sensors and Actuation Technologies in Exoskeletons: A Review

**DOI:** 10.3390/s22030884

**Published:** 2022-01-24

**Authors:** Monica Tiboni, Alberto Borboni, Fabien Vérité, Chiara Bregoli, Cinzia Amici

**Affiliations:** 1 Department of Mechanical and Industrial Engineering, University of Brescia, Via Branze, 38, 25123 Brescia, Italy; monica.tiboni@unibs.it (M.T.); cinzia.amici@unibs.it (C.A.); 2 Agathe Group INSERM U 1150, UMR 7222 CNRS, ISIR (Institute of Intelligent Systems and Robotics), Sorbonne Université, 75005 Paris, France; fabien.verite@sorbonne-universite.fr; 3 Institute of Condensed Matter Chemistry and Technologies for Energy (ICMATE), National Research Council (CNR), Via Previati 1/E, 23900 Lecco, Italy; chiara.bregoli@icmate.cnr.it

**Keywords:** actuators, assistive devices, exoskeletons, rehabilitation, sensors

## Abstract

Exoskeletons are robots that closely interact with humans and that are increasingly used for different purposes, such as rehabilitation, assistance in the activities of daily living (ADLs), performance augmentation or as haptic devices. In the last few decades, the research activity on these robots has grown exponentially, and sensors and actuation technologies are two fundamental research themes for their development. In this review, an in-depth study of the works related to exoskeletons and specifically to these two main aspects is carried out. A preliminary phase investigates the temporal distribution of scientific publications to capture the interest in studying and developing novel ideas, methods or solutions for exoskeleton design, actuation and sensors. The distribution of the works is also analyzed with respect to the device purpose, body part to which the device is dedicated, operation mode and design methods. Subsequently, actuation and sensing solutions for the exoskeletons described by the studies in literature are analyzed in detail, highlighting the main trends in their development and spread. The results are presented with a schematic approach, and cross analyses among taxonomies are also proposed to emphasize emerging peculiarities.

## 1. Introduction

Exoskeletons and exoskeletal robots are wearable devices based on a mechanical structure that conceptually mirrors the skeletal structure of a limb or of the involved body-part. In recent decades and still today, they are the subject of much research, considering the advantages that they can bring to the end-user.

These devices can be classified according to various aspects. Classifications can be based, for instance, on the purpose, on the body part to which they are addressed, on the used actuation technology, or on the type of interaction with the user. Additionally, human–robot interaction through robotic exoskeletons can be aimed at different purposes, and four main classes can be identified: rehabilitation, assistance, performance augmentation and haptic interaction [1].

Robotic rehabilitation provides repetitive, flexible and customizable exercises that complement physiotherapist work, aimed at the functional recovery of patients who report impairments or disorders deriving, e.g., from stroke, brain or spinal cord injuries (SCI), amyotrophic lateral sclerosis (ALS), orthopaedic surgery, or cerebral palsy (CP) [2]. Further significant advantages are associated with the use of robotic devices in rehabilitation, including, e.g., intense repetitive training, performing at home rehabilitation with remote control, automatically adjusting the device support based on the patient progressive recovery [3,4], increasing the patient engagement through computerized activities proposed in the form of games, monitoring progress through the assessment of outcomes in an objective way [5] and reducing the overall cost of rehabilitative care given the aging of our society [6,7].

Assistive robotic exoskeletons can be effectively used to help the elderly or permanently injured in carrying out the most important activities of daily living (ADLs) with more independence, attempting to compensate for disabilities or partial functional loss [8]. Walking, grasping and handling objects and eating are some of these core activities.

In the context of work or military duties, which involve very intense stresses to the human skeletal and muscular structures, the use of an exoskeletal robot can lead to a significant improvement in the operating conditions and a reduction in the physical injuries risks associated with these types of tasks [9]. Specifically designed exoskeletons for performance augmentation combine the strength of a robot with the intelligence of a human to perform tasks that could hardly be done either by a man alone or robot alone. Furthermore, haptic interfaces intended for augmented or virtual reality applications [8] can be developed through wearable exoskeletons.

Considering the anatomical district to which the exoskeleton is aimed, we identify devices for the upper limb (upper limbs exoskeletons ULE), for the lower limb (lower limbs exoskeleton LLE), for the whole body or for a specific anatomical district. A dedicated class is often considered for the hand (hand exoskeletal devices HED), due to the fundamental role that it plays in the ADLs. Likewise, the trunk is often considered individually, given the particular characteristics (such as high amplitude) of the associated movements. For each one of these classes, the adopted design solutions are strongly influenced by the exoskeleton’s purpose.

**Figure 1:**
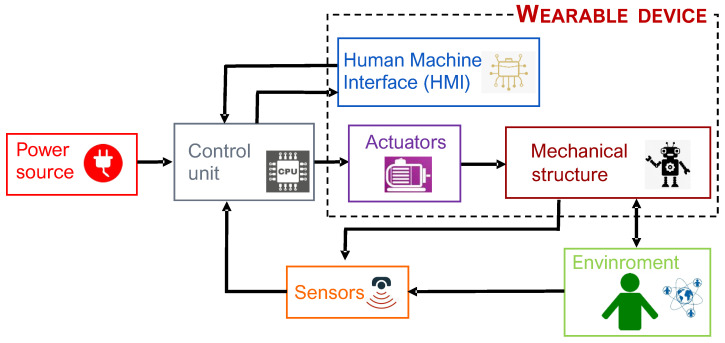
General architecture of a robotic exoskeleton. Actuators and sensors are fundamental elements within the overall system that constitutes the exoskeleton. Actuators are key elements in active devices, and the adopted technological choices significantly influence the device performance. Sensors are essential for the interaction with the environment, in particular with the user of the exoskeleton. The choices for actuator and sensor technologies are often correlated.

Similarly to any other type of robot, a robotic exoskeleton is a complex system of interrelated parts. As the block-diagram of Figure 1 depicts, the following set of fundamental elements can be typically identified:

- a mechanical structure, with degrees of freedom (DoF) consistent with the robot’s purpose;
- actuators, which generate the required mechanical power;
- one or more sources of energy;
- proprioceptive and exteroceptive sensors, providing information on the machine functional status and on the interaction with the user and/or the environment;
- a control unit, processing the signals transmitted by the sensors and instructing the motor controllers;
- human/machine interface(s) receiving information/instructions from users (either the therapist or the user) and providing online feedback; and
- the environment.

The environment, purpose and working conditions strongly affect the requirements that a device is expected to fulfill [10]. For instance, traditional industrial robots that operate in a structured environment can rely on the design philosophy “stiffer is better”, as the main requirements are high precision and speed. Unlike these, a wearable exoskeleton operates in an unstructured environment and must guarantee as main features safety comfort for the user, easy control and low encumbrance [11]. These different design specifications significantly influence the choice of system components and related technologies [12,13,14,15,16].

In the mechanical design, a fundamental choice concerns the number of DoF of the system, that is the sum of all independent movements (i.e., translations/linear displacements and rotations) that can be performed in all the joints of the device [17]. The number of DoF is defined in order to determine the exact position and orientation of all segments of the device. As the number of degrees of freedom increases, the mechanical complexity grows as well, but the handling possibilities also increase. The DoF of the device may be active or passive, whether they are actuated or not. An exoskeleton may have all the DoF active, only some of them or none.

In the first two cases, it is defined an active device and as a passive device in the latter. Finally, we have two further definitions of devices: haptic and coaching devices. Haptic exoskeletons interact with the user through the sense of touch, and their main function is not to cause or to resist movement, but rather to provide the user with tactile sensation.

Coaching devices are non-actuated devices that do not generate any forces but provide different feedback; they can serve as an input interface for interaction with games in virtual reality, such as systems using video-based motion recognition (e.g., Kinect^®^, Microsoft, Redmond, WA, USA).

The energy is provided by the power source. The control unit, based on the indications coming from the human–machine interface (HMI) and on the data acquired by the sensors, decides how the actuators must be powered [3]. This control unit block therefore contains, in addition to a microprocessor that manages the command logo, electrical components that actually provide power to the actuators. The control unit must detect the user intention, provide safe movements and should be structured to favor the portability of the device. Actuators provide motion and forces or torques to the mechanical system.

The set of mechanical system and actuators constitutes the wearable device. Sometimes the HMI devices are an integral part of this device, and sometimes they are external, as well as for the sensors and the control unit. This depends on the structure of each device. The device interacts with the environment, which defines the limits to the movement and imposes boundary conditions for the functioning, such as the rehabilitation strategy, the operating modes and the methods for evaluating the results.

The actuation units can be placed either distally or proximally with respect to the ground of the kinematic frame, and the position of the motors affects the dynamics of the system. The strategy of placing the actuators directly at the joint level avoids the need of a transmission mechanism but increases the inertia of the moving parts, resulting in a less transparent control and in a more power-consuming system. With the choice of proximally placed actuators, a transmission mechanism is required to transmit the torque at the distal location. This reduces the inertia at the joint but also introduces the challenge of compensating for the nonlinear dynamics that may arise in the transmission, such as hysteresis and friction.

Another design choice that significantly influences many fundamental aspects, such as the structure, weight, energy consumption and performance, concerns the technology for the generation of mechanical power. A wide variety of actuation technologies have been used to develop exoskeletal robotic devices. These can be classified according to the nature of the energy source used to generate mechanical power: hydraulic, pneumatic and electric actuation are the most common.

Similarly, different technologies are used for sensing purposes in exoskeletons, such as measures relating to the movement of specific limbs [18], forces or torques exchanged between device and user, bio-signals, such as electromyography signals (EMGs) [19], electroencephalography signals (EEGs) and mechanomyography signals (MMG) to be used for device control or validation [20,21,22], etc.

The mechanical structure (and consequently DoF and concerned body-part), actuation, sensors, HMI, control strategy and purpose are the main aspects on which a review of the research activity on exoskeletons may be focused. Several review articles on exoskeletons have been published in recent years, and those taken into consideration are collected in Table 1. Most of them concern devices dedicated to a specific body part, i.e., the upper or lower limbs, and, among the more recent publications, only the review of Agarwal et al. [1], published in 2019, addresses exoskeletons in a more general way.

The authors, in the first part of their work, analyze the state of the art of exoskeletons used for medical applications and for performance augmentation; in the second part, they examine the sub-components, that is the mechanical design, actuation, sensing, materials and control; in the third part, they describe two case studies, Harmony (for shoulder and upper limb rehabilitation) and Maestro (for hand rehabilitation) and finally discuss ongoing challenges and future directions. The review papers of Sanjiuan et al., in 2020 [17], of Rehmat et al., in 2018 [23], of Blank et al. [24], of Manna et al. [25] and of Maciejasz et al. [26] dated 2014, concern upper limb rehabilitation.

Sanjiuan et al. focused their work on construction solutions based on cable transmission. Rehmat et al. conducted a systematic review on the use of robotic exoskeleton systems for upper limb rehabilitation deepening typical mechanical structures and control strategies for exoskeletons in clinical rehabilitation conditions. The work of Blank et al. deals with robotic stroke rehabilitation for upper-limb therapy focusing on patients engagement. Manna et al. focused their review on a comparative study of actuation systems. Maciejasz et al. developed an extensive and thorough survey on devices for upper limb rehabilitation, including the analysis of over 120 devices. A greater number of reviews in recent years concern exoskeletons for the lower limbs.

Hussain et al. [27] in 2021 proposed a review of materials, actuation and manufacturing methods in exoskeleton robots for lower limb assistance. Shi et al. [28] examined the topics of gait analysis and mechanical design, actuation and control of lower limb exoskeletons. Sanchez-Villamañan et al. [29] reviewed the mechanical design principles of compliant lower limb exoskeletons. Al-Shuka et al. [9] covered biomechanical modeling, actuation and multi-level control strategies of power augmentation lower limb exoskeletons.

Zhang et al. [30] systematically reviewed the developments of robotic lower-limb rehabilitation after stroke, providing a classification, a comparison and a design overview of the driving modes, training paradigm, control strategy and gait perception. Louie et al. [31] proposed a scoping review with the aim of mapping the use of robotic exoskeletons for gait rehabilitation in adults. Chang et al. [32] reviewed the lower-limb exoskeletons to restore gait for individuals with paraplegia. Only one recent review devoted to current hand rehabilitation technologies was found [33], dated 2012.

Within this framework, the current work differs from all the most recent reviews, as we aim at providing an in-depth analysis of the literature about exoskeletons (i) without limitations related to the involved anatomical district and (ii) deepening the most recent trends on both implemented sensors and actuation technologies.

Compared to Agarwal’s work, the proposed review conducts a more extensive investigation of the scientific literature classifying the documents at different levels to extrapolate significant indications on the trends of research activity in this area. Furthermore, the analysis of actuation and sensing techniques is developed with a greater level of detail, as the review particularly focuses on these two aspects. The current work presents an approach similar to the one proposed by Maciejasz et al., in their review of 2014 [26]; nevertheless, the range of interest is here more extensive, as we aim at exoskeletons in general and not only at those for upper limbs, and the literature analysis is updated to date.

The final aim is to trace a picture of the main solutions of exoskeletons, as complete as possible, for all the different uses and for all the different parts of the body. In detail, the proposed literature review presents two different analysis levels: a prospective review, or an observational study of the publication distribution over time, by purpose, by involved body district, by device type, focus and design solutions; and an analytical review, consisting of an in-depth study of the more recent and most used technologies for exoskeleton actuation and sensors.

**Figure 2:**
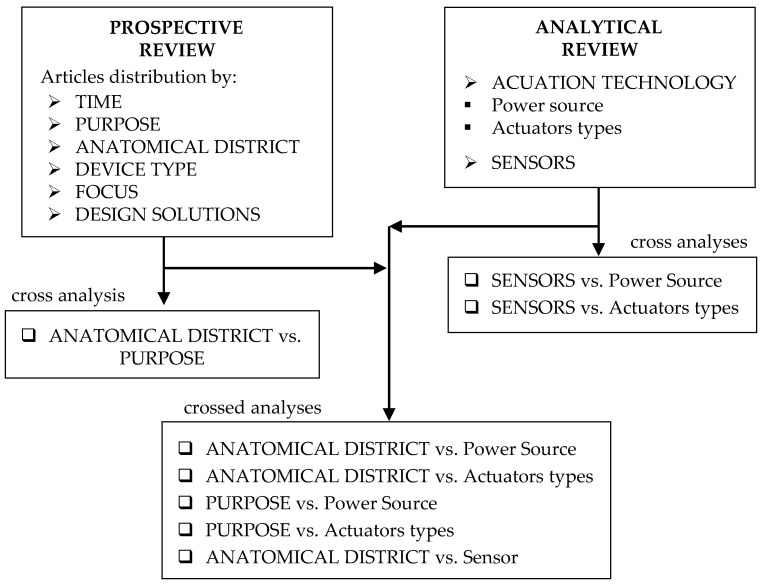
Schematic summary of the review work: prospective and analytical review structures and performed cross analyses details.

Furthermore, cross-analyses were carried out to highlight possible correlations between the investigated issues. The identification of these correlations can provide useful information on the technological choices made for the different types and uses of exoskeletons, as well as on the results obtained with these choices. Figure 2 schematically shows the two main levels of analysis (prospective and analytical) and the cross analyses.

An investigation on technical solutions for exoskeleton design choices with a particular focus on sensors and actuation technologies can be a useful contribution to future projects, highlighting what has already been considered and allowing useful insights from the successes but also from the shortcomings of other works. The aim of the review work is, therefore, to summarize the most recent and widespread solutions for exoskeleton development, which may be a valuable source of information for engineers, physiotherapists and exoskeleton developers in their activity.

The paper is organized as follows: Section 2 describes the research method in terms of the data selection protocol, perspective and analytical review. Section 3 reports the results of the prospective review, whereas Section 4 presents the results of the analytical review, declined for actuation and sensing technology. Section 5 (conclusions) summarizes the more significant findings of the review work.

## 2. Materials and Methods

This section describes the procedure applied for the data selection and depicts the taxonomies adopted for the prospective and analytical reviews.

### 2.1. Data Selection Protocol

The literature analysis was performed querying the Scopus database. To identify the documents related to exoskeletons and particularly focused on sensors and actuation technologies, a search string was designed to ideally detect the documents presenting in the title the fundamental words *exoskeleton* and *sensors* or *actuation*. To capture possible lexical variations, such as the words *biosensors* or *bio-signals*, *motors* and *exo-skeletons*, the final search string was defined as: “TITLE (exo*skelet* AND (*sens* OR actuat* OR *signal* OR motor*))”.

The results were filtered according to the following inclusion criteria: (i) only documents written in English language are considered and (ii) documents must be classified, according to the Scopus database, within at least one of the Subject Areas *Engineering*, *Computer Science*, *Medicine*, *Materials Science*, *Neuroscience*, *Health Professions*, *Multidisciplinary*, *Nursing* and *Psychology*.

**Figure 3:**
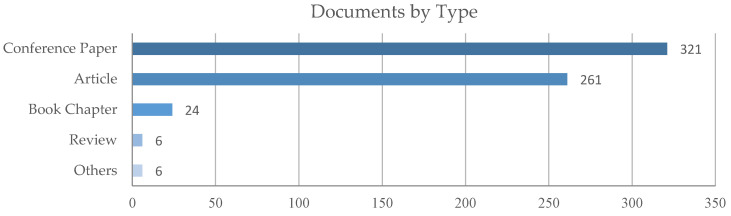
The distribution of the identified set of documents by document type. About 52% are conference papers and 42% are articles, and each of the other categories is below 4%.

**Figure 4:**
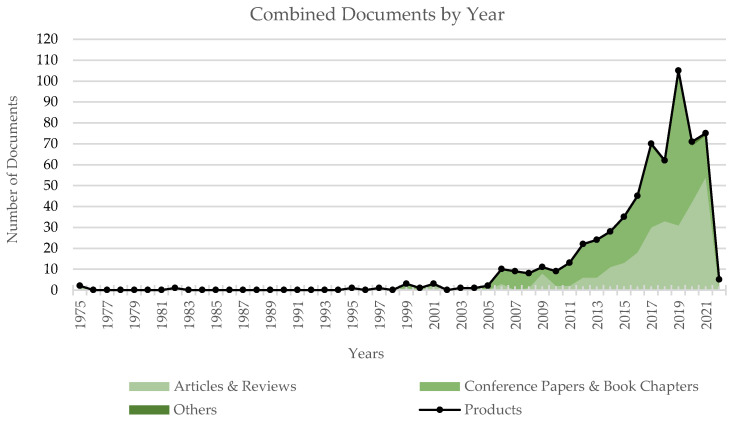
The distribution of the identified set of documents by year among combined categories of documents. Data are presented in stacked format. In the black line with dots is the total amount of documents by year. A significant production of conference papers on the subject started in 2006 and of journal articles in 2009.

The query was updated for the last time on the 7 December 2021 and provided 618 research products. Figure 3 depicts the result distribution among the different document types; products classified as Letter, Note or Erratum were collected in the class Others. Figure 4 describes instead the distribution of documents by year for the following combined classes: Articles and Reviews, Conference Papers and Book Chapters and the previously defined Others. The analysis of the time trend for these classes highlights the growing presence of articles and reviews in the last years.

Therefore, aiming at capturing, at best, the most relevant technological trends depicted in the literature and considering the scientific relevance of articles and reviews in the research context, a further inclusion criteria was applied to the identified results: (iii) only products classified as *article* or *review* document type according to the Scopus database were considered.

**Figure 5:**
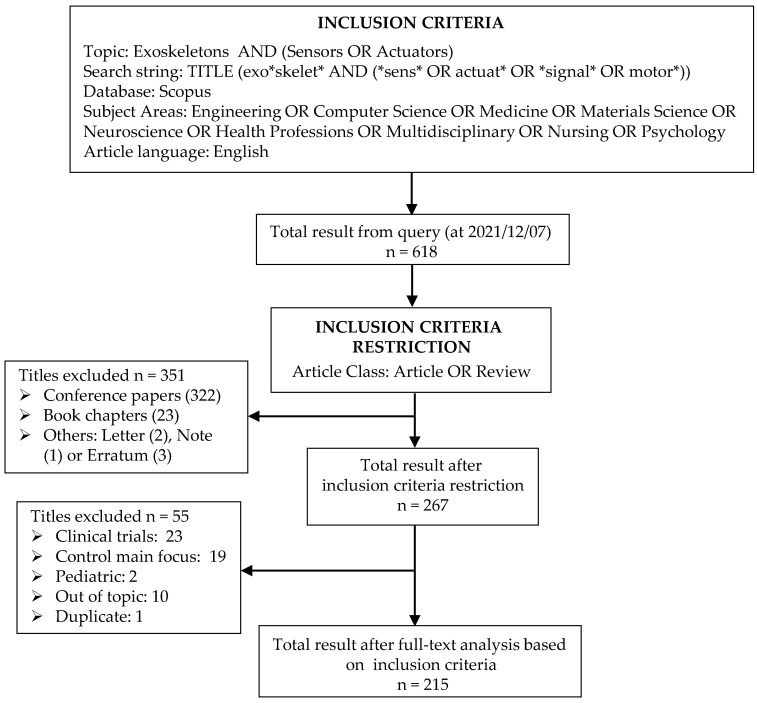
Flow chart mapping the selection process with the number of records identified, included and excluded and the reasons for exclusions.

A further check was finally performed on the 267 emerging products looking for residual formal errors, i.e., inconsistencies with the imposed inclusion criteria and off-topic results, i.e., inconsistency of the document content with the review purpose. Figure 5 provides a flow chart of the selection process, which maps out the number of identified records, included and excluded and the reasons for exclusions. Among the documents found with the query, a significant number (23) falls within a purely medical field, reporting the results of clinical trials with the use of exoskeletons, and another important number (19) concerns, in a very specific way, issues related to device control techniques. As these issues are beyond the scope of the review, the corresponding documents were excluded. Some works dealing with topics completely outside the field of analysis, related to the pediatric field, or duplicates were also excluded. At the end of this evaluation, a final dataset of 215 documents emerged.

### 2.2. Taxonomy

The 215 documents in the final dataset were evaluated according to two analysis levels: a first observational study of the documents distribution over time, by purpose, by involved body district, by power source and by design solutions and a detailed study of the more recent and most used technologies for exoskeleton actuation and sensors. These two analyses enabled the outline of a prospective review and of an analytical review of the literature.

The final taxonomies were the results of a two-phases process that guided the identification and selection of the possible classification items. In a first phase, the full papers of the review articles on the topic were thoroughly analyzed, and, based on this analysis and on authors experience, an initial definition of fields and sub-fields emerged. Subsequently, the first set of items was iteratively updated in the course of the analysis of the full papers: some changes were made, adding some fields that had not been considered or removing others when the items were not dealt with in any work or in a very limited number of publications. The final set of elements considered for each taxonomy and used to analyze all the 215 documents of the defined subset is described in the following.

#### 2.2.1. Prospective Review

The documents were evaluated with respect to the following aspects:

- the **purpose**, meant as the final application envisioned for the exoskeleton by the document’s authors;
- the **anatomical districts**, i.e., the anatomical districts involved by the exoskeleton;
- the **device type**, the presence or absence of actuation;
- the **focus**, defined as the main topic addressed by the paper; and
- the **design solutions**, or peculiar characteristics of the exoskeleton from a technical perspective.

For each aspect, a dedicated taxonomy was designed. The classification of the documents among categories is not exclusive, meaning that the same document can be assigned to more categories within the same aspect.

For the analysis by purpose, the following categories were considered:

**p1**  Rehabilitation/Medical applications. This category gathers exoskeletons designed or developed to be applied in a clinical context, such as rehabilitation training, surgery and tele-operations.
**p2**  Assistive device. Exoskeletons working as ADLs assistive tools are assigned to this category. Devices described by the document’s authors as orthoses are included as well.
**p3**  Power/Performance augmentation. This category collects the exoskeletons devoted to the enhancement of the human power or performance, regardless of the application context (e.g., both industrial and clinical).
**p4**  Haptics. This category identifies the exoskeletons developed with the primary aim of providing feedback signals. Exoskeletons realized as measurement devices or as haptic interfaces are examples of this kind of systems.

For the analysis by anatomical districts, four categories were considered:

**ad1**  Upper Limb, with the subclasses describing the involved body parts:
  **ad1.a**  shoulder,
  **ad1.b**  elbow,
  **ad1.c**  wrist and
  **ad1.d**  forearm.
**ad2**  Lower Limb, presenting the three subclasses:
  **ad2.a**  hip,
  **ad2.b**  knee and
  **ad2.c**  ankle.
**ad3**  Hand, with three subclasses:
  **ad3.a**  single finger,
  **ad3.b**  more fingers and
  **ad3.c**  wrist.
**ad4**  Trunk.

Particular attention was devoted to the analysis of the category ad3 hand. In fact, this category was investigated in terms of anatomical districts simultaneously involved by the device, thus, allowing the evaluation of the exoskeleton from a functional perspective.

The analysis by device type includes the following categories:

**dt1**  Actuated device. The documents in this category present exoskeletons with at least one active DoF. For exoskeletons enabling rehabilitation training, a further distinction is also performed between:
  **dt1.a**  Passive rehabilitation. Exoskeletons in this category allow performing passive rehabilitation, i.e., the rehabilitated body part of the user is moved by the device, without contribution provided by the user themselves.
  **dt1.b**  Active-assisted rehabilitation. In this second kind of rehabilitation, the user is required to actively move the device. Exoskeletons in this category can assist the patient during the motion, providing the user with additional force, when needed.
**dt2**  Unactuated device. Exoskeletons presenting only passive DoF are classified within this class.
**dt3**  Coaching device. Exoskeletons in this category are explicitly described as coaching systems or devices to support measurement and training.

In the analysis by focus, three categories were defined:

**f1**  Sensors. Documents assigned to this category describe innovative sensors applied to exoskeletons, related experimental setups or measurement systems.
**f2**  Actuation system. This category includes papers mainly devoted to the description of motors and actuation technologies from the design of new components to the investigation of unconventional solutions based on traditional elements.
**f3**  Other. This category collects the documents that deal with exoskeletons and sensors or actuation technologies but present, as a main focus, a different topic. For instance, papers principally describing clinical trials, modeling, control methods or algorithms for signal processing are classified in the category f3 Other.

In the analysis by design solutions, a set of technical characteristics was evaluated. The following categories were identified:

**ds1**  Transmission. This category investigates the solutions implemented to transform the motion of the actuators and to actuate the joints. The class presents six subclasses representative of the adopted technology:
  **ds1.a**  Cables, as flexible cables or wires.
  **ds1.b**  Gear/Screw, including gearboxes and worm gears.
  **ds1.c**  Belt.
  **ds1.d**  HD (Harmonic Drive).
  **ds1.e**  Direct transmission.
  **ds1.f**  Linkages or cams.
**ds2**  Portable device, describing whether the exoskeleton is defined as a portable system.
**ds3**  Joining. This category analyzes the connection strategy between user and machine. Four subclasses were identified:
  **ds3.a**  rigid interfaces,
  **ds3.b**  latches,
  **ds3.c**  velcro or strap-based systems and
  **ds3.d**  other solutions, such as braces or air cushions.

In addition to those categories, other characteristics were investigated, such as the number of active and passive DoF of the exoskeletons and the custom or commercial nature of the device.

#### 2.2.2. Analytical Review

For the analytical review, the dataset was classified according to a double taxonomy. In particular, implemented actuation technologies and sensors were investigated.

For the analysis by actuation technology, four main categories of power supply were defined:

**A1**  Electric actuation, with the subclasses:
  **A1.a**  DC motors,
  **A1.b**  SEA (series elastic actuators),
  **A1.c**  brushless,
  **A1.d**  induction,
  **A1.e**  VSA (variable stiffness actuators),
  **A1.f**  torque motor,
  **A1.g**  linear motor and
  **A1.h**  stepper.
**A2**  Pneumatic actuation.
  **A2.a**  PAM (pneumatic artificial muscles) and
  **A2.b**  soft-actuators.
**A3**  Hydraulic and Electro-hydraulic actuation.
**A4**  Others.
  **A4.a**  SMA (shape memory alloys),
  **A4.b**  EAP (elaectroactive polymer) and
  **A4.c**  magneto-rheologic fluids.

In the analysis by sensors, the following categories were selected:

**S1**  Bending Sensors, such as flexion sensors.
**S2**  Dynamic Sensors, able to capture dynamic quantities, including torques and forces and including the subclasses:
  **S2.a**  pressure sensors,
  **S2.b**  torque sensors,
  **S2.c**  force sensors and
  **S2.d**  Inertial Measurement Unit (IMU) sensors.
**S3**  Electromyographic (EMG) Sensors.
**S4**  Electroencephalographic (EEG) Sensors.
**S5**  Cameras, or optical-based systems.
**S6**  Encoders.
**S7**  Other Sensors, including for instance potentiometers.

### 2.3. Data Analysis

According to the described taxonomies, the 215 documents of the final dataset were analyzed and mapped in dedicated tables (see Table A6 in the Appendix A). The categories in each classification have not been treated as exclusive classes, meaning that the same document could be mapped in more categories depending on the presence of specific characteristics. Data were then rearranged and evaluated, and the main results are presented in the following with a schematic approach.

## 3. Prospective Review

**Figure 6:**
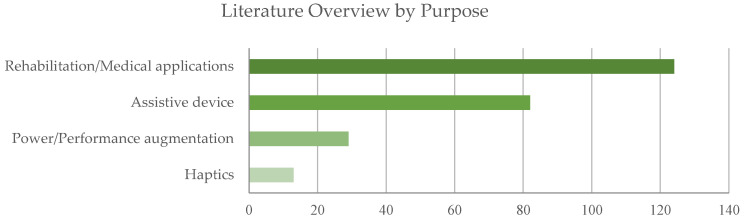
The distribution of exoskeletons by purpose. Studies on exoskeletons for rehabilitation (or more generally for medical applications) and assistive devices cover the 58% and the 38%, respectively.

**Figure 7:**
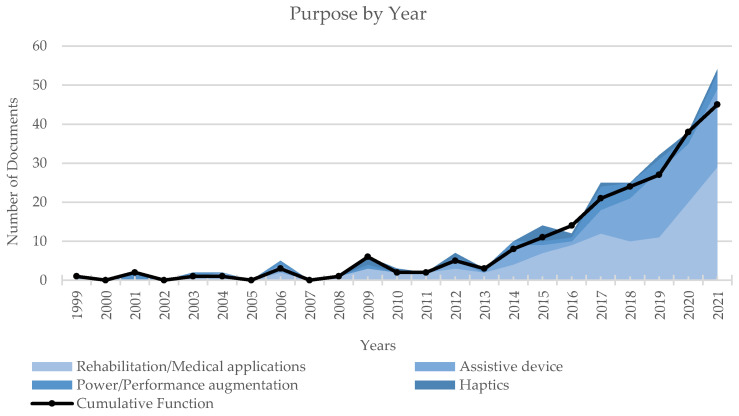
The distribution of the identified documents by year, among categories of the taxonomy by purpose. The data include all the occurrences and are presented in stacked format. In the black line with dots is the total amount of documents by year. A growth trend in the scientific production of articles for medical applications occurs since 2013, and, around 2019, the growth rate of related papers increases.

For the prospective review of the literature, different aspects were considered, i.e., purpose, focus, anatomical districts, device type and design solutions. To evaluate the literature by purpose, the selected documents were analyzed in terms of distribution among categories as a whole (Figure 6) and by year (Figure 7). The data reveal that most of the exoskeletons were conceived for medical applications, whereas only a limited number of devices were envisioned as haptic systems.

The predominance of papers in the category p1 rehabilitation/medical applications emerges both in terms of absolute numbers and in terms of the relative relevance of this category with respect to the others by year. The trend shown in Figure 7 reveals a significant increase in research activity on exoskeletons for medical applications since 2019.

**Figure 8:**
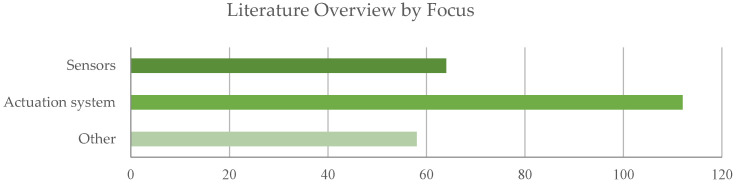
The distribution of exoskeletons by focus. About 52% of the revised publications have the actuation system as main focus and 30% concern sensors. The Others category includes jobs for which there is a significant part related to the actuation or sensors, but the main focus is different (design, kinematics, modeling, validation or signal processing).

**Figure 9:**
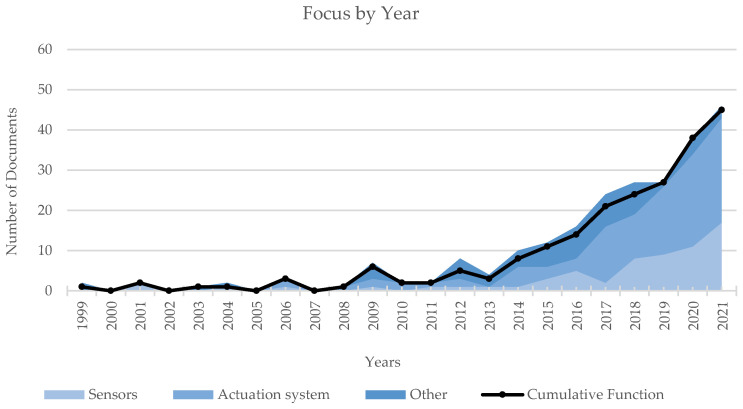
The distribution of the identified documents by year, among categories of the taxonomy by focus. Data include all the occurrences and are presented in stacked format. In the black line with dots is the total amount of documents by year.

The analysis of the dataset by focus is synthesized in Figure 8 and Figure 9, describing the documents distribution among categories in aggregated form and by year. Both the graphs reveal a remarkable interest of the scientific community towards the actuation system (f2). This trend could be expected if considering that actuator dimensions and weight are still critical elements of the overall design of exoskeletons. In addition, the proposed classification strategy could penalize the number of papers actually classified in the category f1 Sensors. In fact, several works focus on algorithms, data fusion techniques and control strategies, particularly among the most recent literature: those contributions, though, are primarily assigned to the category Others (e.g., [34,35,36,37,38,39,40,41,42,43,44,45,46,47]).

The trends of Focus by Year (Figure 9) and Purpose by Year (Figure 7) show that 2013 marked the beginning of a growth in scientific production with an almost constant slope; the same year also identifies the starting event in the growth of works on exoskeletons for rehabilitation/medical purposes, whereas the growth phase for assistive devices only begins in 2016. In addition, 2016 also marks the beginning of a greater research in actuation systems. These last two trends are most likely correlated, as portable assistive devices require more energy-efficient, more compact, less heavy and more dynamically performing actuation systems.

**Figure 10:**
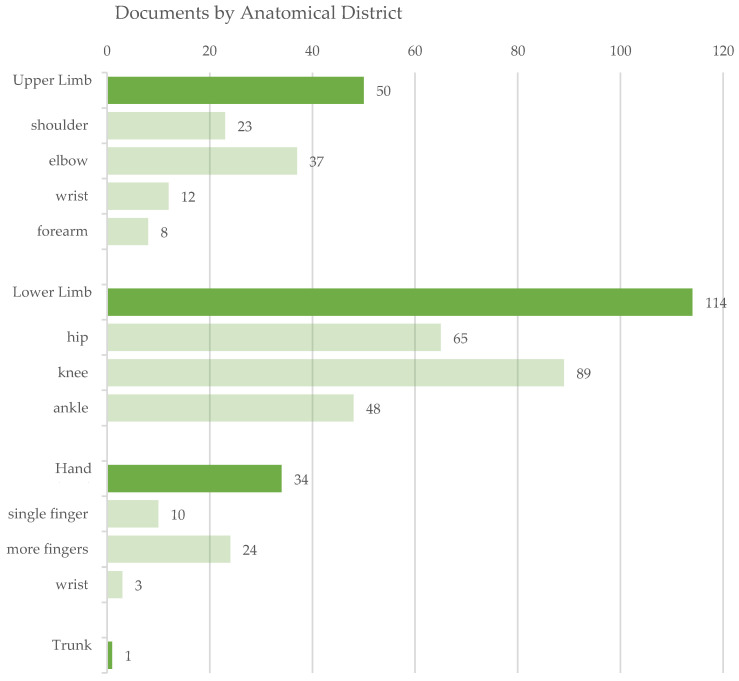
The distribution of exoskeletons by anatomical districts. The 57% of revised articles deals with lower limb exoskeletons and knee devices are dominant. For the upper limbs, the elbow is the most considered joint and multiple fingers for the hand.

The analysis by anatomical districts highlights the main presence of exoskeletons devoted to the lower limb and to the knee joint in particular. As Figure 10 depicts, the lower limb category collects more than twice the number of exoskeletons for the upper limb. This behavior could be expected, given that the functional movements of the lower limb are less complex than the ones assured by the upper limb, involve cyclic and well known tasks (e.g., gait) and given that damage or impairment at the lower limb strongly affects the subject’s quality of life.

For those devices, some design challenges still unresolved are regarding the dimensions and portability of the system, battery time-span and user comfort. For the upper limbs, exoskeletons must face different technological challenges, for instance to deal with the anatomical complexity of the shoulder joint. In this analysis, the wrist was considered in both the upper limb and hand category, but each contribution was classified in one of the two subclasses depending on the focus of the exoskeleton.

The hand was considered an independent anatomical district with respect to the upper limb according to a functional rationale: in fact, given the different functional role of hand and upper limb, exoskeletons are also more commonly devoted to the treatment of one of those categories. The hand offers peculiar anatomical conditions, such as non-negligible effects due to soft tissue artifact [48]; this introduces specific technical challenges, for example in the joining at the finger level, that require dedicated solutions (e.g., digits).

For this reason, particular relevance is given in documents to the possibility of multi-fingers treatments, and often particular attention is paid to the management of the thumb, independently as in the works by Agarwal et al. [49] and Wang et al. [50], or with other fingers (e.g., [51,52,53]). The trunk is specifically addressed in the paper by Ko et al. of 2018 [54], although the stabilization of the trunk is considered in clinical practice to be a fundamental step and a pre-requisite for the enrollment in several rehabilitation training paths.

**Figure 11:**
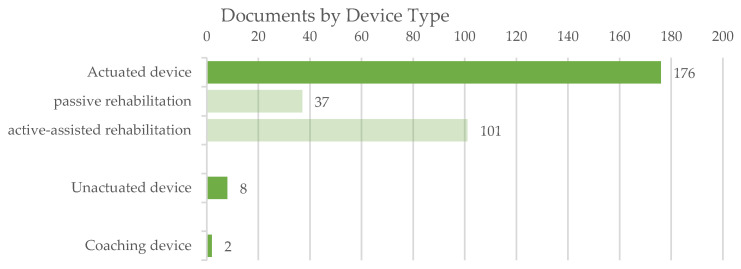
The distribution of exoskeletons by device type. The majority of the scientific work concerns actuated exoskeletons, and active rehabilitation is the most frequent.

The analysis of the documents by device type allows discriminating among exoskeletons that enable passive or active-assisted rehabilitation. Figure 11 synthesizes the distribution of the devices in the different categories; actuated exoskeletons cover almost the full amount of devices. In this classification, some exoskeletons could be mapped in more categories; this happens when the device is explicitly described as working according to different configurations (e.g., as an actuated or unactuated device).

**Figure 12:**
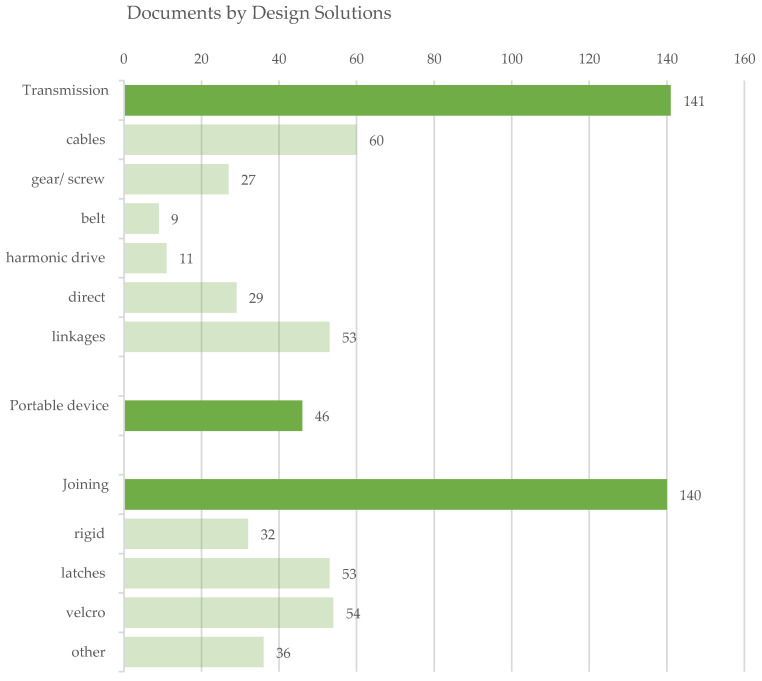
The distribution of exoskeletons by design solutions. Among transmissions, cables and linkages are prevalent with similar percentages values (around 40%). Latches and velcro are almost equally used, with a percentage of about 38%.

Evaluating the exoskeletons by design solutions (Figure 12), most of the documents explicitly refer to transmission and joining details or allow capturing hints about the adopted technical solutions. For the transmission, most of the exoskeletons are grounded on cable-based systems; however, linkages are also a spread solution to connect rigid bodies of the kinematic chain and assure the motion transmission. For the joining between human and machine, different solutions are presented, but the most commonly adopted are strap-based systems. In the subclass ds3.d other, braces and gloves are often used, but rubber and foam are also adopted, integrating the rigid connections [53,55,56,57,58,59,60,61,62,63].

**Figure 13:**
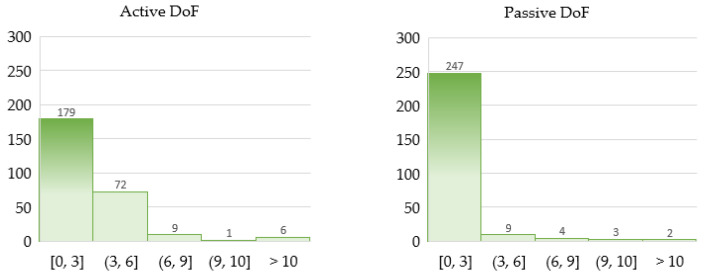
The distribution of exoskeletons active and passive DoF. A limited number of degrees of freedom is preferred both in the case of active and passive ones, but for the active ones, devices are more frequent with DoF between 3 and 6.

Portability of the exoskeletons is a technical characteristic analyzed in about the 20% of the documents, whereas a dedicated analysis can be performed on active and passive DoF. As Figure 13 depicts at a glance, from a general perspective, actuated DoF are less frequent than passive joints, and a lower number of DoF is preferred for both active and passive DoF. These trends fit well with the tendency towards simple and compact design strategies. Nevertheless, opposite solutions, which favor DoF redundancy despite the kinematic complexity, are also present; those solutions tend to ease complementary factors, such as the control management of the device.

**Figure 14:**
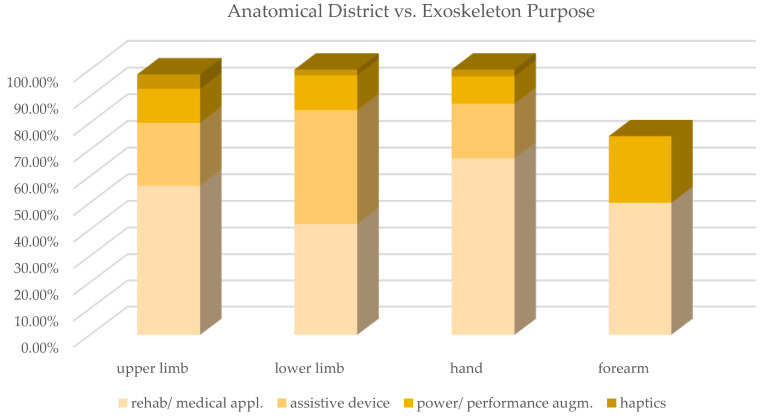
The distribution of exoskeletons purposes for the exoskeletons dedicated to the different anatomical districts. Rehabilitation/medical applications are dominant for ULE (56%), hand (67%) and forearm (50%) devices. For LLE they are almost equally frequent as assistive devices (about 42%).

**Figure 15:**
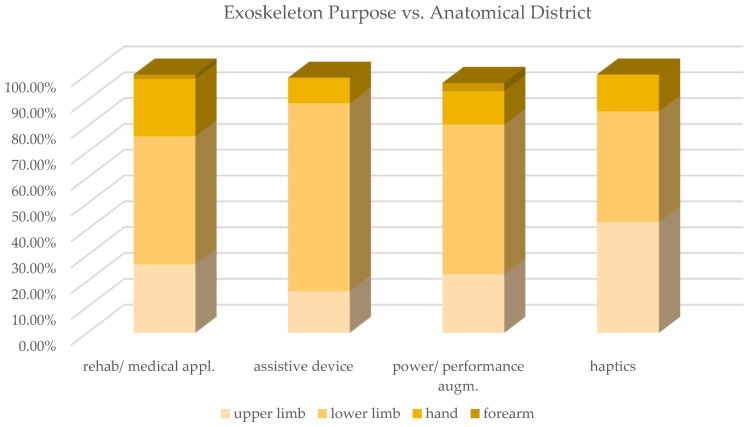
The distribution of the exoskeletons dedicated to the anatomical districts for the different exoskeleton purposes. For Rehabilitation/medical applications, LLE are prevalent (50%), as well as for power/performance augmentation exoskeletons (58%) and even more for assistive devices (73%).

For purpose and anatomical districts, a cross analysis was performed, and the classification results are reviewed in Table A1 and Figure 14 and Figure 15. The trunk district was not included in this cross analysis due to the very limited number of contributions. Rehabilitation/medical exoskeletons are predominant for ULE, hand and forearm exoskeletons, while assistive devices are predominant for LLE. Allowing paraplegic people to regain the ability to walk is an important challenge that has led to intense research on assistive devices for the lower limbs. The growing demand for robotic devices for elbow, hand and shoulder rehabilitation and, in particular, for portable devices that allow home therapy, is motivated by our aging society.

The increase in people’s life expectancy leads to growth in post-stroke patients affected by hemiparesis; this explains the prevalence of devices dedicated to this purpose for the upper limbs and the hand. The power/performance augmenting exoskeletons are developed for all the anatomical districts, with a very similar percentage between ULE (12.73%), LLE (13.04%) and hand (10.26%); the percentage reaches 26% in haptic exoskeletons, but this is not a significant value given the small number of devices. Analyzing the distribution of exoskeleton purpose vs. the anatomical district, it emerges that the predominance of LLE is confirmed regardless of the purpose of the device, and, in assistive devices, it has the highest percentage (as noted above).

## 4. Analytical Review

To perform an analytical review of the literature, an evaluation of the documents with respect to the specific aspects of sensors and actuation technologies was performed.

**Figure 16:**
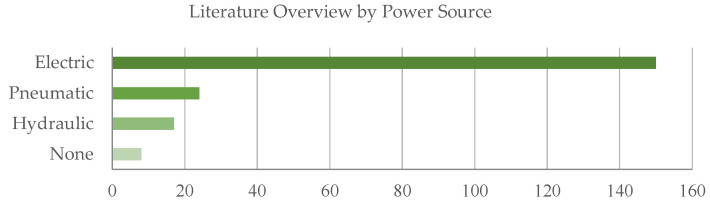
The distribution of exoskeletons by power source. Electric power source (73%) is undoubtedly the most used. Pneumatics is adopted in the 13% of the revised works and hydraulics in 9%.

**Figure 17:**
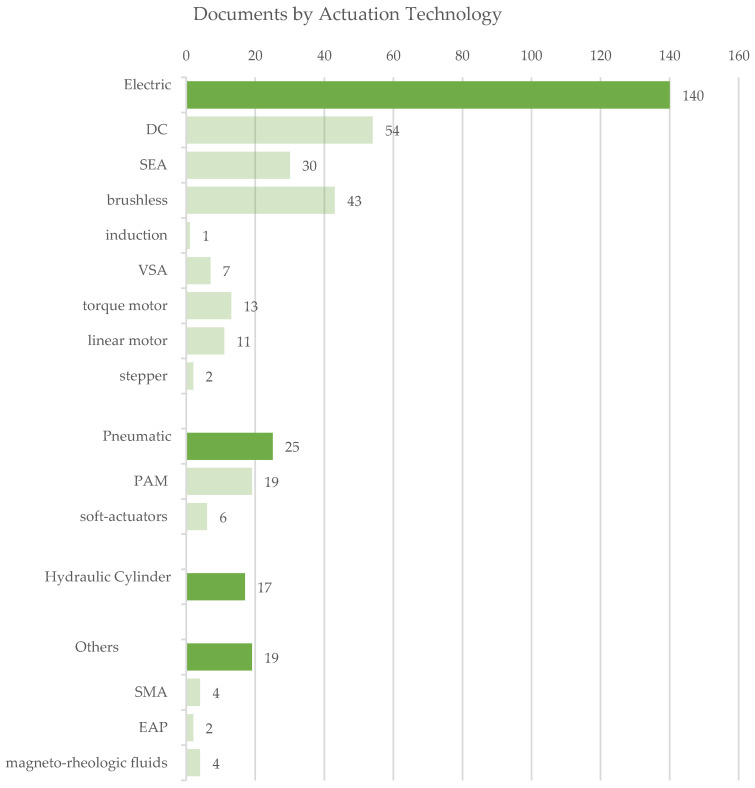
The distribution of exoskeletons by actuation technology. Among the electric solutions, brushed and brushless DC motors are the most used (39% and 31%, respectively). SEAs are also quite widespread (22%). Pneumatic solutions are mainly developed with PAMs.

**Figure 18:**
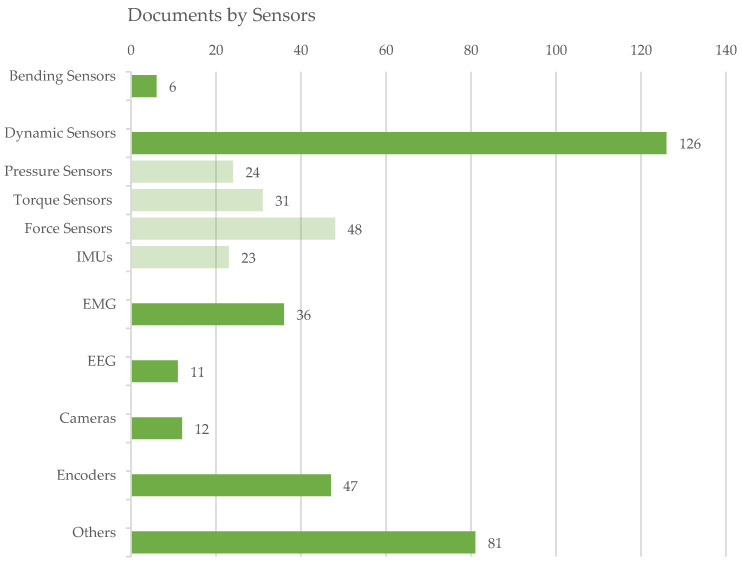
The distribution of exoskeletons by sensors. In the sensors for exoskeletons context dynamic sensors are predominant (40%), with a significant number of force and torque sensors. Encoders are also quite diffused (14%). The Other class has a relevant weight (25%), because a wide range of different other types of sensors are used within exoskeletons.

For the analysis of the actuators implemented in the identified exoskeletons, Figure 16 presents the detected power source by type. An electric power source represents the most common solution, whereas pneumatic and hydraulic solutions follow. The final class None collects the exoskeletons without external power actuation. Figure 17 depicts the presence of references to the specific subclasses in the analyzed documents at a glance. Likewise, Figure 18 synthesizes the occurrences of the different kinds of sensors in the evaluated dataset.

The following sub-paragraphs show the results and the related discussion with reference to the two main topics of investigation of the review: the actuation systems and the sensors. For the first topic, specific subsections are dedicated to the electric, pneumatic, hydraulic and electro-hydraulic actuation as well as to the aspects related to the motion transmission that have a close connection with the chosen actuation technique. For the sensors, dedicated insights were made for bending sensors, dynamic sensors, EMGs, EEGs, cameras and optical vision systems, encoders and other sensors.

### 4.1. Actuation Technologies

In the proposed analytical review, the term actuation refers to the subsystem that generates the mechanical power at the joint level, and therefore, in this discussion, the session power source, actuator type and transmission design solutions are included. Exoskeletons actuators should act similarly to biological muscles and their neuro-mechanical control, as for all robots that strictly interact with people; for this reason, new constructive solutions of actuators have been developed specifically for this application field.

**Figure 19:**
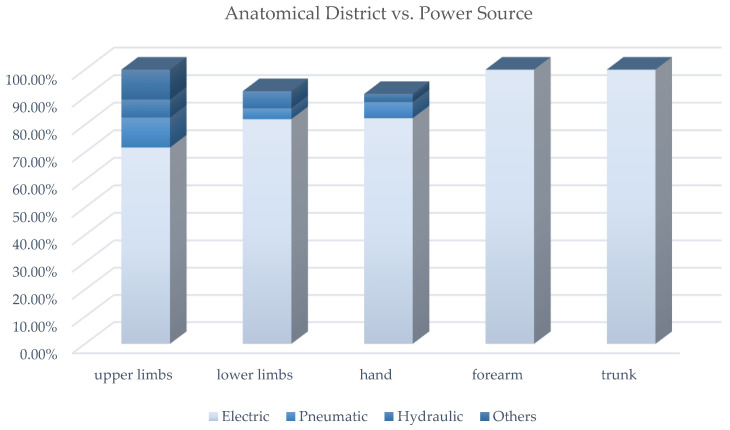
The distribution of power source technologies for exoskeletons dedicated to the different anatomical districts. Electric technology predominates regardless of the anatomical district to which the exoskeleton is dedicated (72% for ULE, 82% for LLE and hand exoskeletons). ULE pneumatic actuation is at 10%, and other solutions have the same percentage.

Electric, pneumatic and hydraulic are the available types of power sources. The choice of the power source has a fundamental role in the device design as it influences the final system main functional characteristics, e.g., the overall dimensions, weight, stiffness, autonomy, back-drivability, control accuracy, forces and torques that can be generated. Table A2 contains a cross-analysis of the paper distribution of power source vs. anatomical district. Figure 19 shows how power source technologies are distributed in exoskeletons dedicated to the different anatomical districts.

**Figure 20:**
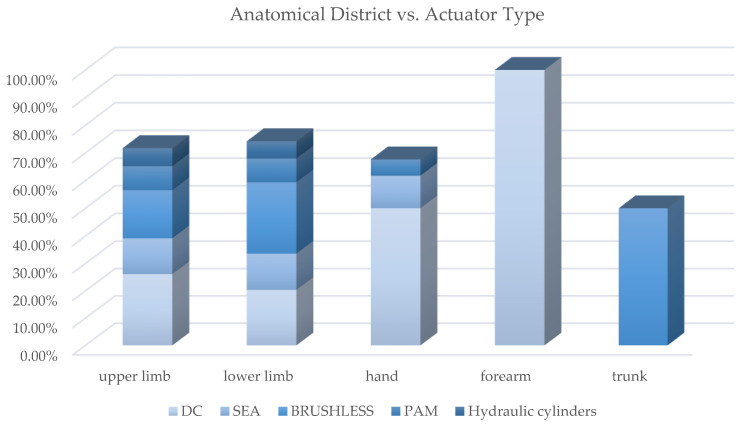
The distribution of the most used actuator types for exoskeletons dedicated to the different anatomical districts. Brushed DC motors are dominant for hand exoskeletons (50%), have percentages of 26% in ULE and 21% in HED (Hand Exoskeletal Device). Brushless DCs are dominant in LLE (26%), are present with the 17% in ULE and are not present in HED. PAMs have percentages of 8%, 8% and 6% for ULE, LLE and HED, respectively.

**Figure 21:**
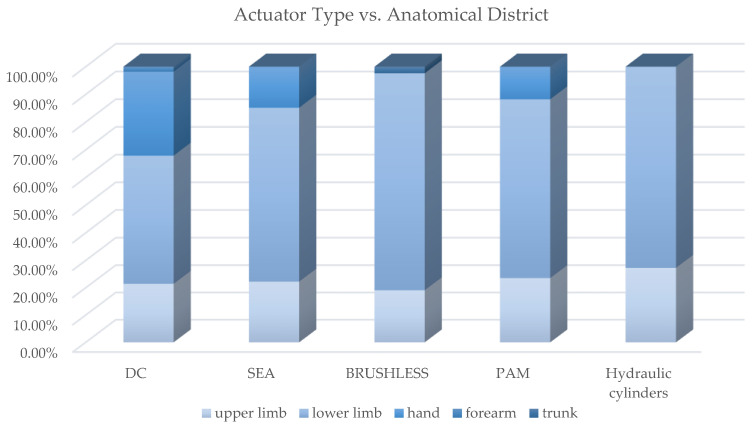
The distribution of the anatomical district to which the exoskeleton is devoted for the most used actuator types. For all the actuator types, included in the histogram LLE is the dominant application with a lower percentage (46%) for brushed DCs, for which HED have also a significant weight (30%).

Figure 20 shows how most used actuator types are distributed in exoskeletons dedicated to the different anatomical districts; Figure 21, instead, reports for each one of the most used actuators the kind of exoskeleton, depending on the dedicated anatomical district, in which it is used. The following deductions emerge from the observation of the table. The crossed analysis confirmed what was already well known [1,26], i.e., most of the designed and developed devices are electrically operated.

This predominance of electric actuation is independent on the anatomical districts to which the exoskeletons is aimed at. Pneumatic and hydraulic actuation, instead, are mainly adopted in lower limb exoskeletons, while particular actuators, such as SMA, EAP or magneto-rheological fluids are very little used and never for LLE. Brushed and brushless DC motors are the most used electric actuators, and, in recent years, SEA are increasingly used.

**Figure 22:**
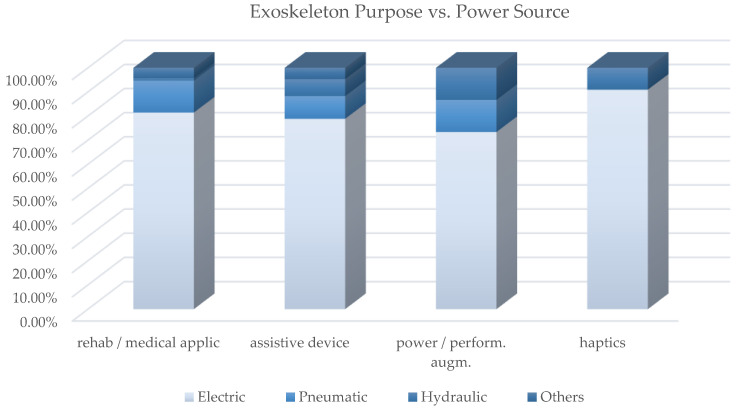
The distribution of the power source technologies for exoskeletons with different purposes. Electric actuation is the predominant choice regardless of the exoskeleton purpose (81%, 79%, 73% and 91% in the displayed order). Pneumatics has about 13% in medical applications and power/performance augmentation EDs (Exoskeletal Devices), and, in these last devices, hydraulics have a similar percentage (13.3%).

**Figure 23:**
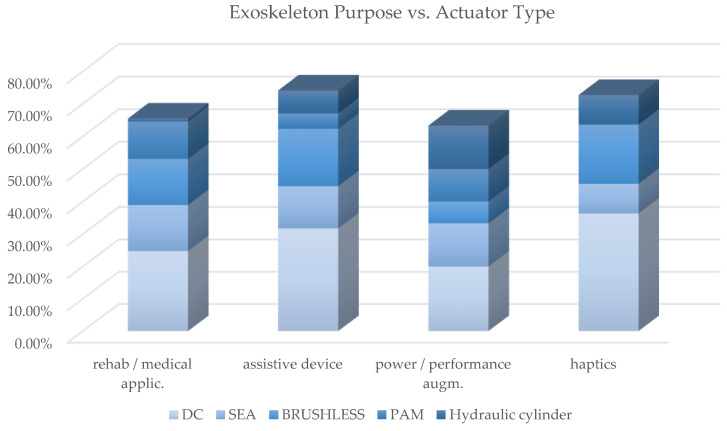
The distribution of the most used actuator types for exoskeletons with different purpose. The brushed DC motor is the most frequent choice for all the EDs purposes (25%, 32%, 20% and 36%, in the displayed order). For medical EDs SEAs, brushless and PAMs also have a quite similar weight (14%, 14% and 12%). Brushless in assistive devices have quite significant diffusion (18%). Hydraulic cylinders have a percentage of 13% in EDs for power/performance augmentation.

**Figure 24:**
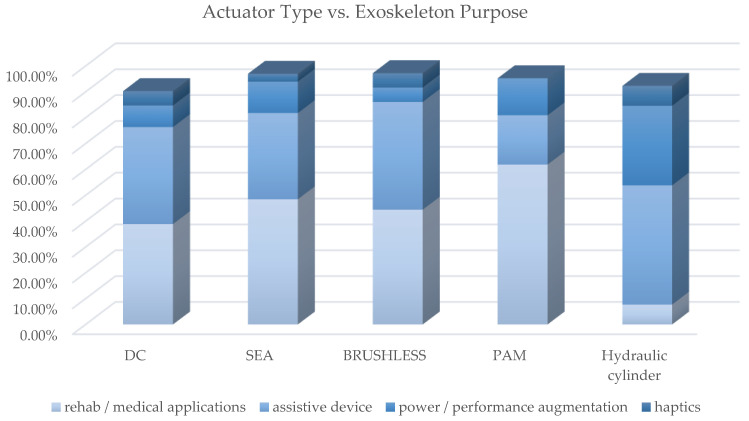
The distribution of the exoskeleton purpose for the most used actuator types. Medical and assistive purposes have similar weights for the most used actuators: DC, SEA and brushless motors without a clear prevalence of one over the others. For PAMs, rehabilitation/medical applications are clearly dominant (62%).

Table A6 contains the cross-analysis of the paper distribution of power source vs. exoskeleton purpose. Figure 22 shows how power source technologies are distributed in exoskeletons with different purpose. Figure 23 shows how most used actuator types are distributed in exoskeletons with different purpose; Figure 24 reports, for each of the most used actuators, the kind of exoskeleton, depending on the purpose, in which it is used.

For the most adopted electric actuators (DC, brushless and SEAs) an equal distribution between rehabilitation and assistive exoskeletons is observed. Instead, VSAs, torque and linear motors, which are overall little used, are mainly diffused in medical or for rehabilitation prototypal devices. Even the PAMs are mainly used in exoskeletons for rehabilitation, while hydraulic actuation is mainly used in assistive devices or for power/performance augmentation. The following paragraphs analyze, in detail, the main trends for the exoskeleton development for the different possible alternatives in terms of the power source.

#### 4.1.1. Electric Actuation

Electric solution for actuation is widely used because it allows easy energy storage and supply. Electric actuators are available in a great variety of types and sizes, are reliable and allow a good control accuracy. Among the different types, brushed DC electric motors are the most widely used, followed by brushless BLDC motors. The brushed DC motor is used, in many cases, because of the simple structure of the motor itself and of its electronic drive; however, the brushes and commutator system require regular maintenance.

Therefore, the main advantages of brushless vs. brushed motors are the greater reliability, due to the lack of brushes and the better dynamic performance, allowed by a lower rotor inertia and the higher power-to-weight ratio. In some cases, direct drive torque motors, which are placed at the joints, are used. Correct actuators selection and sizing is a key step in the exoskeleton design to obtain lightweight and transparent systems.

Calanca et al., in [64], presented a methodology based on a graphical tool that matches actuator capabilities to the task requirements, thus, showing how different design choices affect the actuator as a whole. In the proposed approach, task torques and velocities are acquired through experimental trials, repeated by different subjects; a motion capture system allows the acquiring of position and velocities, while joint torques are estimated via inverse dynamics on a multi-body human-exoskeleton model.

Once these datasets are available, the procedure guides the component selection and sizing. Similarly, Barjuei et al., in [65], proposed an approach to the selection of a brushless BLDC motor and a gearbox transmission based on an optimization through a human–robot dynamics interaction analytical model and a mathematical relation between the weight and technical characteristics of the components. The optimization criteria are expressed in terms of the closed-loop system frequency bandwidth, system power consumption and the weight of the components and are formulated by imposing technical constraints on simulation parameters.

In some cases, the actuators or the reduction units are of non-standard design; therefore, the need to characterize them arises. Belogusev and Egorov, in [66], presented a quick and inexpensive method for determining the efficiency of a electric actuation system, which allows higher-precision measurements. The same authors in [67], proposed an automatic measurement process for determining the starting torque of an electric gear actuator for an exoskeleton. The method does not require expensive equipment and can be performed without dismounting the actuator from the exoskeleton.

A key feature of an exoskeleton is in not hindering the wearer’s movements to achieve comfort and safety; therefore, the joints must be back-drivable. Several authors focused on this aspect, such as Liu et al., in [68], who tested the active joints of the developed upper-limb power-assisted exoskeleton and proved their excellent back-drivability. Safety and back-drivability requirements for exoskeleton actuation systems have favored the research and development of new electric actuation solutions, e.g., Series Elastic Actuators (SEA), Variable Stiffness Actuators (VSA), Parallel Elastic Actuators (PEA) and Magneto-Rheological Series Elastic Actuators (MRSEA). Series Elastic Actuators (SEAs) [49,52,63,69,70,71,72,73,74] are formed by a spring, or a spring-like component, in series with an electric actuator (usually a BLDC or a DC motor).

The springs ensure that the coupling between the user and the motor be compliant, thereby, protecting the users body from impact loads and other undesirable interactions. Furthermore, the compliance introduced by the spring facilitates a torque-based control strategy by transforming the torque/force control problem into a position control problem based on the measurement of the springs deformation. These actuators, widely used in exoskeletons, allow a smooth force transmission, accurate force control, lower output impedance, shock tolerance, energy efficiency and back-drivability in human–robot physical interactions.

The spring acts as an impact damper and reduces the actuator inertia felt by the user, thus, allowing the user to increase safety and comfort. A further advantage is the peak motor power reduction exploiting the spring capacity of storing energy. The main disadvantages of SEAs are the reduction of the positioning bandwidth and the rise in the number of mechanical parts with a possible consequent overall weight increase. To improve the exoskeleton dynamics when SEAs are used, Vantilt et al., in [72], presented a novel model-based torque control, based on extensive modeling of the exoskeleton and of its interactions with the environment. Various other issues on exoskeletons with SEAs are dealt with in the literature.

Aguirre-Ollinger and Yu, in 2021 in [69], proposed a force feedback control for a lower-limb assistive exoskeleton driven by variable-structure SEAs coupled via Bowden cables and proved its stability. The SEAs variable structure refers to the effect of the commanded force that allows varying the stiffness between two levels. Marconi et al., in 2019 in [52], presented a novel series-elastic actuators (SEA) architecture, for a hand exoskeleton that directly measures externally transferred torque in real-time and, thus, enables both position- and torque-controlled modes of operation, allowing implementation of both robot-in-charge and user-in-charge exercise paradigms.

Hsieh et al., in 2017 in [73], proposed a shoulder exoskeleton with linear SEAs to obtain accurate force and impedance control at the exoskeleton–limb interface. A variant of SEA is represented by SEAC [75], series of elastic actuators with clutch, in which a mechanical clutch automatically disengages the transmission when needed. This mechanical solution improves actuator transparency and safety and guarantees the desired assistance in terms of both timing and torque magnitude.

Other very promising actuators for exoskeletons belong to the class of Variable Stiffness Actuators (VSAs), which is a sub-class of Variable Impedance Actuators (VIAs). VSAs are inspired by the human capability to adapt the joint stiffness to the external conditions and perturbations and can vary their mechanical impedance directly at the physical level as the natural musculo-skeletal system does. These actuators may change the stiffness without the need of an active control capable to simulate different stiffness values. Safety, energy-efficiency and resilience are the main advantages associated with the use of VSAs.

Schrade et al., in [76] developed a lower-limb exoskeleton, the VariLeg, with a variable mechanical stiffness actuation (VSA) unit that drives the knee joint. This solution allows a low energetic cost of transport of human walking. Additionally, adjustable compliance is also expected to increase efficiency, safety and stability of human–robot interaction in gait rehabilitation. The authors also demonstrated that such adaptable compliance provides advantages to cope with uneven terrain or external perturbations and increases the achievable gait speed by allowing more dynamic walking.

Liu et al., in [77], tested the stability robustness test of a variable stiffness actuator (VSA) programmed with the Gain Scheduling-based Variable Impedance Control (GSVIC). The proposed control system follows the paradigm of the variable impedance task in accordance with human intention. Cestari et al., in [78], presented an actuator with Adjustable Rigidity and Embedded force Sensor (ARES) that is conceived as a force-controlled variable impedance actuator. This actuator not only provides elasticity on the joint but also allows intrinsically performing a measure of the torque exerted by the joint. Some authors have developed exoskeletons in which both SEAs and VSAs are present. Baser et al., in [70], presented the lower limb exoskeleton named BioComEx with one variable stiffness actuator (VSA) for the ankle and two series elastic actuators (SEA) for knee and hip joints.

Recently, another solution of compliant actuator was developed: the Parallel Elastic Actuators (PEAs). Penzlin et al., in 2021 in [79], investigated the parallel elastic actuators (PEA) and postulated that the efficiency of such drives in cyclical motion tasks, such as gait, can be increased by employing an elasticity acting in parallel to the actuator. Toxiri et al., in [80], proposed PEAs for assisting workers in carrying and lifting weights while reducing the actuator’s peak electrical torque and accelerating its reaction.

Another SEA variation is the Magneto-Rheological Series Elastic Actuator (MRSEA), which takes the advantages of both SEA and MR brake. Chen et al., in [71], presented a MRSEA designed for the knee joints of a lower extremity exoskeleton. The actuator, by reducing the overall mechanical impedance of the exoskeleton, filters out unwanted collisions and, furthermore, improves the system energy efficiency by providing large controllable braking torque with low power.

A particular actuation solution that deserves to be mentioned is that proposed by Han et al., in [74]. Han presented a multimodal actuator, based on a motor paired with a clutch (at the center) and brakes acting in parallel, which allow the output to be actuated or passive. The multimodal actuator is capable of operating a joint with different modes, e.g., a series elastic mode can be used for fast running by storing energy in the spring, or a stiff position actuated mode allows a precise joint control when carrying large payloads.

#### 4.1.2. Motion Transmission

The analysis of exoskeleton mechanical transmissions (Figure 12) revealed that cables are the most adopted solution. Using the cable transmission system results in a significant reduction in the exoskeleton’s weight and in the required torque at the joint level. The cable-driven exoskeletons fall in the so-called flexible exoskeletons category, which has more natural human–machine interaction. At present, among cable transmissions for exoskeletons, Bowden cables are the most common. Bowden cables’ working principle is the transmission of the motor driving torque to the joints through cables, winding wheels and pulleys [81].

Using Bowden cable transmissions, often the motor and transmission are located to the wearer’s back (in particular for LLE) to improve the mass distribution. Very often Bowden cables are used in conjunction with SEAs [52,69,82,83,84,85], as the spring element is connected with the Bowden cable, obtaining the so-called serial elastic actuator Bowden cable drive. Agarwal et al., in 2017 in [49], presented a hand exoskeleton to move the thumb featured by a large range of motion (RoM), based on a series elastic actuation with Bowden cable, allowing for bidirectional torque control of each thumb joint individually.

In [69], Aguirre-Ollinger and Yu investigated a novel force control method for a SEA-driven lower-limb assistive exoskeleton and the SEA is powered by a DC brushless motor, which transmits force to the Bowden cables via the springs. The regulation of the springs deflection is used for the force control, and the Bowden cables exert torque on the exoskeleton joint by means of a pulley and a fixed-axis rotary coupling. An innovative use of cable transmission is presented by Chen et al., in [86], in which two identical SEAs are connected through a novel cable-driven differential that couples the elbow flexion/extension and the forearm supination/pronation joints, thus, allowing actuator load sharing, structural member size reduction and a compact design.

In some cases, the solution named Quasi-Direct Drive (QDD) actuation is particularly suitable [87]. QDD actuation (also known as proprioceptive actuation) is based on a high torque density motor coupled with a low gear ratio transmission and allows high bandwidth and high back-drivability for a wide variety of human activities. Some authors developed solutions based on the reduction in the number of actuators, such as Ko et al., in [54]. In that lower-limb exoskeleton (LLE), with an actuator, through wires and a differential gear mechanism simultaneously, both legs were driven.

#### 4.1.3. Hydraulic and Electro-Hydraulic Actuation

The main advantages of hydraulic actuation are its inherently compliancy, low joint inertia and high loads, while the main drawbacks are related with the resulting in non-portable, heavy and difficult to manipulate exoskeletons in addition to the risk of hydraulic fluid leak. Successful implementations of these actuators are mainly in the LLE systems, for which the load capability is one of the most important requirements. As well as for electric actuators, variable stiffness, magnetorheological clutches and cable transmission concepts are also adopted for hydraulic transmission. Zhu et al., in [88], described the design and experimental testing of a unidirectional variable stiffness hydraulic actuator applied to an exoskeletal knee, in which a variable ratio lever mechanism with linear elastic element was used to achieve the adjustable passive compliance of the joint.

Long et al., in [89], presented a LLE with a pump-based hydraulic actuation system with unidirectional cylinder with embedded springs on the cylinder rod to make the hydraulic actuation system compact and lightweight. The springs help to control the leg in the swing phase without consuming energy of hydraulic system. Khazoom et al., in [90], proposed a LLE system, which combines delocalized magnetorheological (MR) clutches with a hydrostatic transmission using low-friction rolling diaphragms to distribute power around the body to provide transparent and yet powerful multifunctional exoskeleton assistance.

Lu et al., in [91], presented a LLE with a drive system based on hydraulic actuators and tendons drive for each joint, distinguished by high power and low inertia. The proximal end of tendon connects each rod of the cylinder and the distal end of tendon is fixed on the joint. The tendon can move freely in the sheath. The tendon is pulled by the hydraulic cylinder to produce the flexion and the extension in the sagittal plane.

Often, an electro-hydraulic drive is the preferred choice. Staman et al., in [92], presented the design, control and evaluation of LLE exoskeleton for gait restoration developed with PREHydrA (passive return electro-hydrostatic actuator). The goal was to develop a high force density actuator, using remote actuation to relocate mass to favorable locations to improve the wearable aspect. The PREHydrA concept was shown to produce high output forces over a range of frequencies relevant to wearable robotics.

The design of an electro-hydraulic actuator (EHA), which has both the hydraulic and electric advantages, was treated by Lee et al., in [93]. The EHA system consists of a hydraulic bidirectional pump, a motor, a hydraulic cylinder and the valves obtained in a manifold. The motor attached to the pump allows the hydraulic actuator position and speed control. This solution provides high level of power, but the hydraulic circuit simplicity reduces manufacturing costs and allows for easy leakage manage.

#### 4.1.4. Pneumatic Actuation

Pneumatic actuators have intrinsic compliance and a high power-to-weight ratio but limited forces and torques values—features that make them suitable for exoskeleton actuation when high forces or torques are not required. As confirmed by the cross analysis synthesized in Table A2, PAM is the most widely adopted pneumatic actuator in exoskeletons. PAM is formed by a not expandable double-helix-braided shell wrapped around a rubber tube. When the tube is inflated with pressurized air, this causes a PAM contraction in the longitudinal direction, and, when it is deflated, it returns to its original shape; therefore, it is an alternative actuator.

Compared to electric motors, PAMs have several advantages, e.g., inherent compliance, low cost, a high power-to-weight ratio and compactness. In addition to these advantages, the PAM operation is characterized by hysteresis and significant nonlinearity; therefore, appropriate control strategies must be developed to improve PAM performance (e.g., the accuracy of joint trajectory tracking) and to obtain effective actuation solutions for soft exoskeletons. Furthermore, PAMs are unidirectional, and thus to obtain bilateral rotations, they must be used in pairs, i.e., two PAMs are mounted in an antagonistic configuration.

The PAM control problem was addressed by Cao et al., in [94], who used an artificial neural network, an echo state network [95] to approximate the dynamics of a PAM-driven exoskeleton with a nonlinear autoregressive exogenous model to forecast its behaviors. The same authors, Cao and Huang in [96], used nonlinear model predictive control (NMPC) and an extension of the echo state network called an echo state Gaussian process (ESGP) to design a tracking controller for a PAM-driven lower limb exoskeleton. Zhao and Song, in [97], introduce a novel proxy-based sliding mode control (PSMC) to obtain an accurate trajectory tracking.

A PAM variant is the Pleated PAM (PPAM), developed at Vrije University Brussels. Beyl et al., in [98], for a LLE used PPAMs with a novel design that allows for a higher torque range in a larger range of motion. Cable-driven transmission, as well as with electric actuators, is also used with PAMs. Chen et al., in [99], presented the design, dynamic modeling and motion control of a cable-driven ULE actuated by PAMs. In order to perform passive rehabilitation exercises, dynamic models were developed, and an adaptive fuzzy sliding mode control was designed for the rehabilitation trajectory control.

Hybrid solutions with pneumatic actuators acting in parallel with electric ones have been proposed. Chakarov et al., in [100], presented an exoskeleton with hybrid electric-pneumatic actuation, in which the pneumatic drive takes care of the initial reaction of the force, and the electric drive complements the pneumatic drive. Aguilar-Sierra et al., in [101], described a LLE in which two types of actuators are applied: DC motors with the harmonic drive and PAMs. The hybrid actuation overcomes the short-comings of the two kinds of actuators, e.g., low control accuracy and modeling difficult due to the pneumatic artificial muscle, compactness and structural flexibility of DC motors.

Other types of soft-actuators used in exoskeletons have been developed and tested. Zhang et al., in [102], introduced the design of a vacuum-actuated rotary actuator applied to a wearable soft knee exoskeleton that aids active knee motions during walking. Oguntosin et al., in [103], demonstrated the design, production and functional properties of an Exoskeleton Actuated by the Soft Modules (EAsoftM).

The soft modules, developed to be attached to the joints, are made of polyethylene and have multi-cells with one end glued together by thermal adhesion. ABS frames were used to maintain the structure of each cell when deflating the modules by applying negative pressure. The inflatable actuator was pressurized by pneumatic actuators through silicon tubes, and, as the individual cells start to push against each other, the whole module produces the torque in the fan-shaped manner as a function of pressure.

### 4.2. Sensors

As schematized in the block diagram of Figure 1, sensors are an indispensable element in an exoskeleton, as they allow detecting information from the environment (that includes the user) with which the robot interacts. These collected data are essential to control the exoskeleton. Different types of information may be necessary depending on various aspects, such as the type of exoskeleton (e.g., depending on the anatomical district to which it is intended), the type of control and the mode of operation.

**Figure 25:**
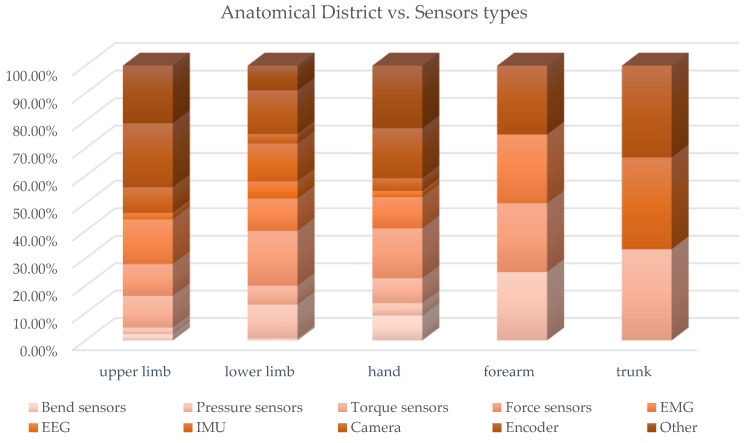
The distribution of the sensor types for exoskeletons dedicated for different anatomical districts. For ULEs, LLEs and HEDs, a significant variety of sensors is used, and for all, there is not one sensor type that is significantly more used than the others. For ULEs, encoders cover the largest percentage (23%); for LLEs, this is force sensors (20%). In HEDs, encoders and force sensors are both present for the 18%.

**Figure 26:**
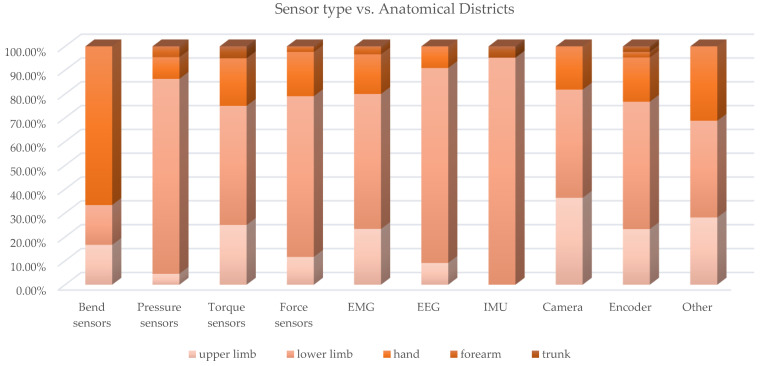
The distribution of the anatomical district to which the exoskeleton is devoted for the different sensor types. For almost all types of sensors, LLE is dominant, particularly for IMUs (95%), pressure sensors (82%) and EEG (82%). Bending sensors are mainly used in HED (67%).

Consequently, many types of sensors can be used, such as force, torque, pressure, EMGs, EEGS and bending sensors, IMU, cameras and encoders to measure physical quantities, such as positions, displacements, rotations, forces, torques, accelerations and muscle and neuronal activations. A cross analysis of the paper distribution of sensor types vs. anatomical district was conducted, and Table A4 collects the paper classification, while Figure 25 and Figure 26 present, in a synthetic way, the results.

As already highlighted by the prospective review, the exoskeletons for the lower limbs are the most widely investigated; consequently, the distribution of the sensors according to the anatomical district also shows the highest concentration of sensors related to the LLE. For this type of exoskeleton, in particular those of the assistive and rehabilitative type, a topic of great importance is gait analysis to recognize the different phases and consequently check, in real time, the contribution provided by the exoskeleton and keeping synchronization with human movements. For this purpose, force, torque and pressure sensors as well as IMU, EMG and encoders are the most used, and very often they are used in a multi-sensor configuration [20].

The sensing of human intention motion is a common concern to many exoskeletons, not only LLE but also ULE or hand exoskeletons. In recent decades, the use of biosensors has become increasingly widespread. These sensors detect biological signals, such as muscular electromyographic, mechanomiographic and electroencephalographic signals. EMG signals have been widely investigated for real-time motion intention recognition from muscle potential activations with sophisticated control algorithms that are sometimes based on machine learning classification methods [104]. Mirroring techniques are used in hemi-paretic subjects to favor a faster neuro-motor recovery [19].

The cross analysis highlights that the dynamic sensors (pressure, force, torque sensors and IMU) are, as a whole, the most used in hand exoskeletons and ULE. The detection of human intention can also be performed by detecting the neuronal activity of the brain through EEG, and the application of this technique for the control of exoskeletons, particularly LLE, was investigated, but there are still many limitations due to the complexity of brain signals. The position feedback (e.g., with linear or rotational encoders) is essential in the functioning with control in force or torque of the compliant actuators and devices that are becoming increasingly widespread for use in robots interacting with humans, SEAs, PEAs, VSAs and MR brakes.

**Figure 27:**
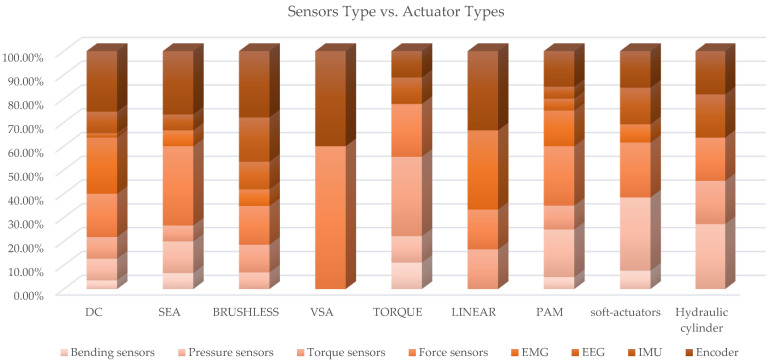
The distribution of the sensor type with the different actuator types. Encoders have an important and almost equal presence with brushed DC motors (26%), SEAs (27%) and brushless motors (28%). Force sensors are widely used with SEAs (33%) and VSAs (60%) and with fluidic actuators. Pressure sensors are highly used with soft actuators (31%), PAMs (20%) and hydraulic cylinders (27%).

**Figure 28:**
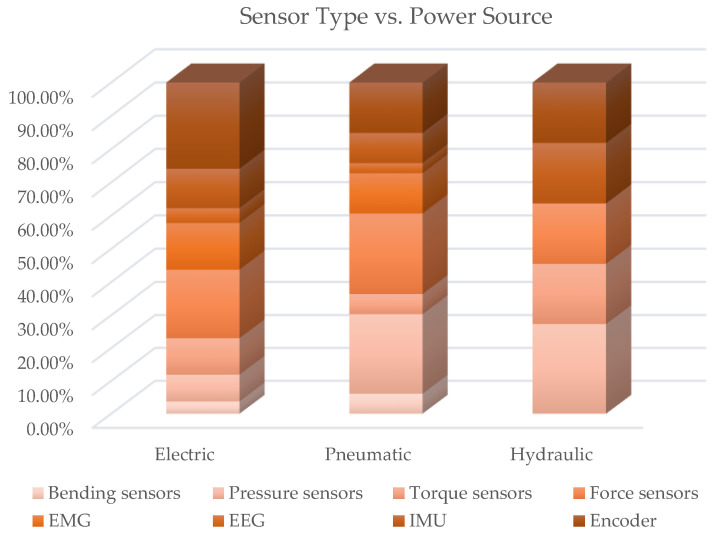
The distribution of the sensor types for the different power sources. With electric actuation, encoder (26%) and force sensors (21%) are the most used; EMGs, IMUs and torque sensors follow with the percentages 14%, 12% and 11%, respectively. With pneumatics, pressure sensors and force sensors are equally frequent (25%), and IMUs follows with 15%. With hydraulics, pressure sensors are dominant with 27%, while force sensors, torque sensors, IMUs and encoders are equally present (18%).

Further cross analysis of sensors vs. actuator types and vs. power source was carried out. Table A5 collects the paper classification, while Figure 27 and Figure 28 present, in a synthetic way, the results. The following considerations emerge from the observation of Figure 27: For exoskeletons with DC, SEA and Brushless actuators, encoder are the most used sensors (with percentages of around 26%); pressure sensors are particularly used when fluidic actuators are present (as is predictable). Force sensors are highly used with SEAs, VSAs and torque motors; torque sensors are used particularly in devices with torque actuators and linear motors.

Bending sensors are somewhat used when soft-actuators and torque motors are present. IMUs are mainly used in exoskeletons with hydraulic cylinders and brushless motors. Analyzing Figure 27, it emerges that, in exoskeletons with electric drives, encoder sensors are predominant, followed by force sensors, EMGs, torque sensor and IMU in that order; with pneumatic power sources, pressure sensors are the most used, followed by force sensors, encoders and EMGs; even with hydraulic actuation, a similar distribution is observed, with the difference that, in this case, IMUs also have a significant weight.

The following paragraphs analyze, in detail, the main class of sensors used in exoskeletons, and we attempt to detect the trends for the sensing issue in exoskeletons.

#### 4.2.1. Bending Sensors

Bending sensors are adopted to measure the characteristics of a deflecting element from a neutral configuration. The main objective of bending sensors is to detect the magnitude and orientation of the bending force produced by a deflecting element; thus, these sensors are used for bending quantification and, indirectly, to measure other physical quantities [43,105], such as pressure and stress, in the exoskeleton and in the interaction with external environment. These elements are particularly interesting in wearable electronic and electromechanical devices, such as active exoskeletons, or to directly measure human motion because they can work in different positions with respect to the human body and are tolerant to different wearing configurations.

These are very promising for applications because of their flexibility to be used in different positions and angles on the exoskeleton and can measure the direct bending or the bending due to pressure from pressing external object. They can measure also a mix of joint movements, such as in finger movements of glove-exoskeletons [106]. A bending sensor is composed of an elastic part and a rigid part. The elastic part is used to restore the deflection of the deflecting element, such as a human finger in the case of finger-exoskeletons [107].

The rigid part of the bending sensor is surrounded by an elastic material. This material can be a rigid or semi-rigid tube, in this case, the bending sensor will be referred as a “Tubular Active Bending Sensor”, or it can be made of a rubber film, such as a “Rubber Active Bending Sensor”. In artificial pneumatic muscles [108], the elasticity of a rubber film can be produced using air pressure. For non-rigid materials, there are several techniques used to deform them: torsion, compression, shear and stretching [62]. The rigid part of a bending sensor is used to transmit the characteristic quantities to the central processing unit (CPU), which directly measures the quantity desired.

It also can be directly connected to an actuation element, which can be a motor or a valve in case of exoskeleton applications. Some bending sensors have been developed for wearable applications and for special sensors for picking and grasping objects. The most adopted technologies are piezoeletric, resistive, optical and capacitive [18]. Piezoelectric effects allow the generation of an electric energy when a piezoelectric element is deformed. Different authors focused their attention on the relation between output voltage and bending curvature.

These sensors can be adopted, i.e., as surface sensors integrated in low flexion actuators. Other piezoelectric sensors considered also the effect of bending speed to improve the sensing accuracy and precision. In this last case, it is possible to measure high bending motions, i.e., in glove exoskeletons. A thick resistive material is coated onto a thicker plastic insulating substrate to create resistive bending sensors [58]. The resistive strip is screen printed with a unique carbon ink on the flexible substrate. With the deflection caused by an applied external force, the resistance value of the ink varies. They can be used as electronic goniometers on body joints [56] to create goniometric clothes for assessing the relative configurations of human body segments.

Optical-fiber bending sensors have benefits over other electrical sensors in terms of small size, anti-electromagnetic interference, corrosion resistance, high sensitivity and cheap maintenance costs; hence, they are frequently utilized in bending measurement. In these devices, a light is emitted from a source and passed through an optical fiber to reach a light-sensitive element. The light passing through the fiber is altered by deflection of the fiber; thus, according to the measuring methodologies used, the optical fiber bending sensor used in exoskeletons may be split into three categories: intensity modulation, wavelength modulation and frequency modulation.

Capacitive bending sensors are based on the relative displacement of two adjacent surfaces that constitute a capacitor. As their distance varies, the capacitance, which is related to the bending, also varies. The variation of the capacitance is proportional to the bending displacement up to a critical range, depending on the geometry of the sensor and on the material used. Beyond this range, the force required to produce a given displacement becomes higher due to increasing flexion and thus displacing contacts. In general, it is used as a simple bending sensor or in touch sensors for human-environment interaction or exoskeleton environment interaction.

#### 4.2.2. Dynamic Sensors

Dynamic sensors, used in exoskeletons, can be divided into four major macro-categories: Pressure Sensors, Force Sensors, Torque Sensors and IMUs, depending on the physical quantity they measure. These sensors are particularly suitable to measure interaction actions between exoskeleton and human limbs. Pressure sensors are adopted in exoskeletons that use fluidic actuators or other parts, such as PAMs or, more in general, soft actuators. The measure of fluidic pressure is indirectly related with other dynamical variables, such as force/torque interaction with external environment and with the subject wearing the exoskeleton.

When high band signals are important, i.e., in real time force control, piezoelectric sensors are usually preferred. Pressure sensors can also be used inside soft-sensors, as in the work by Wang et al. [109], where a wearable, low-cost and compact soft sensor system for direct force measurement in a hip exoskeleton is presented. The sensor is based on a soft pneumatic chamber, in which the dynamic interaction forces are converted into the air pressure changes and measured though a differential air pressure sensor. Different materials and shapes of the chamber were compared to provide useful guidelines in the design of soft sensors.

Force sensors allow the direct measurement of the force exerted by the user at the interface. Force sensors are also used to validate devices or for performance estimation. Usually force-sensors used in this context are load cells. In the work by Kazeminasab et al. [110], load cells are used for the validation of a hand exoskeleton, i.e., for force sensing at the fingertip. The exoskeleton uses SMA actuators and an under-actuated tendon-driven mechanism. The device is capable of exerting extremely high force levels to grasp objects; it can provide 45 N gripping force.

Hamaya et al., in [111], used force sensors to measure the user–robot interaction in a two-degree-of-freedom upper-limb soft exoskeleton with four PAMs. Choi et al., in [112], presented a compact force sensor system with two FSR (force-sensitive resistors) for an assistive LLE sensors. The system measures the assistance force, i.e., delivered force and power of the exoskeleton for motion control and taking urgent safety action. A very peculiar application of force sensors was proposed by Zhang et al., in [113], to develop a spasm sensor to detect joint spasm force with the principle of force detection and identify the spasm type to be applied in hand exoskeleton for rehabilitation.

Torque sensors are mainly used in LLE. Chen et al., in [114], developed a LLE in which each joint is fitted with an absolute encoder, incremental encoder and torque sensor that record the joint angle, angular velocity and torque, respectively, and he proposed a method to predict the human motion intention while walking based on an estimation of the active joint torque of human lower limbs.

Yu et al., in [87], presented a hip exoskeleton composed of a waist frame, two actuators, tow torque sensors and two thigh braces, based on a custom quasi-direct drive actuation (i.e., a high torque density motor with a low ratio gear). An unusual way to detect the torque resulting at the interface user-exoskeleton, and which is presented in various studies, is the use of a SEA actuator as a sensor. Jarrett et Mc Daid in [115], presented a model for an elastomer-based series elastic actuator (eSEA) and tested its feasibility to provide torque sensing as a haptic interface for soft, comfortable HRI in exoskeletons.

As can be seen in Figure 27, IMU sensors are almost exclusively used for LLE; in fact, one of their main functions is to help in the complex task of studying the gait phases. Susanto et al., in [116], used an IMU sensor to recognize the pitch angle generated from the knee joint while the user of a LLE walks as useful information about the walking gait cycle. Often, multiple sensors are used together; an example is given; however, there are many works in which this has been found.

Kim et al., in [105], presented the development of a modular knee exoskeleton system that supports the knee joints of hemiplegic patients. In order to determine the user’s intention, force-sensitive resistors (FSRs) in the user’s insole, a torque sensor on the robot knee joint, and an encoder in the motor are used. In multi-sensor systems, a need that may arise is sensor information fusion.

Qi et al., in [117], proposed an improved greedy reduction algorithm; the data from seventeen sensors in a LLE were collected, and the set theory and the improved greedy algorithm were used to reduce and select the suitable set of sensors. Sun et al., in [118], presented a sensor reduction technique for force/torque sensors utilizing a Kalman filter-based sensor fusion system.

#### 4.2.3. Electromyographic (EMG) Sensors

Electromyography (EMG) is a method of assessing and recording the electrical activity of skeletal muscles. When muscle cells are electrically or neurologically excited, this technique may detect the electric potential emitted by these cells. The signals can be studied to look for anomalies, levels of activation, or recruitment orders as well as to associate it with the kinematics of the generated movement. Two main approaches can be adopted: needle EMG, where a needle connected with an electrode is inserted into the muscle, and surface EMG, where an electrode is attached to the skin in the proximity of the muscle.

In exoskeletons, thsi technique can be adopted to command the motion of the device to combine the motions of the subject and the exoskeleton in an active-assisted approach or to simply evaluate the activation pattern of the subject during the realization of the motion. Needle EMG is an invasive practice and must be performed by experienced medical personnel trained in the use of this procedure, while surface EMG is a non-medical method and, therefore, can be easily performed in various contexts. Furthermore, as the exoskeleton is a wearable system that can be removed from the human subject, there are no needle EMG applications in combination with exoskeletons in the literature.

Needle EMG could be used in the case of prosthesis; although, in these situations, a subcutaneous and fixed electrode innervation is conceivable, avoiding also, in this case, the insertion of a needle through the skin. On the other hand, surface EMG is subjected to various artifacts associated with skin slippage, electrical resistance of the skin and its variability with atmospheric conditions and hydration level. Therefore, needle EMG remains a method for accurate neurological examinations where high measurement accuracy is strictly required. In some of the analyzed works [119,120,121,122,123], EMG was used to collect data on the activation pattern of a subject wearing an exoskeleton.

In the works by Moon et al. [124] and Chandrapal et al. [125], EMG was used for intention detection for a single leg knee exoskeleton, and a similar approach was used in the works by Kiguchi et al. [126] and Rosen et al. [127] for an elbow exoskeleton. In the paper by Aguilar-Sierra et al. [101], the EMG signal of a lower limb was used for command of a lower limb exoskeleton. In Li et al. [128], an EMG signal of the upper limb was analyzed in real-time and adopted in the controller of a lower limb exoskeleton to adjust to the height and width of stairs. In Fleischer et al. [129], the EMG signal was used in two different real-time controlled exoskeletons, a single knee and a hand exoskeleton.

#### 4.2.4. Electroencephalographic (EEG) Sensors

Electroencephalography (EEG) is a representation of the temporal evolution of the electric fields measured on the skull’s surface. The spontaneous oscillations of membrane potentials at the level of brain synapses are represented by the EEG signal, which is generated by neurons on the cortical surface. The EEG can be used to assess spontaneous or induced brain electrical activity in both normal and diseased settings. EEG recordings come in a variety of shapes and sizes, depending on the scenario.

The location of the generators (source) and an electric dipole, which, in turn, depends on the directionality of the ionic fluxes, affects the EEG signal recorded at the surface. EEG signal has only some correlation with the electric brain activity. Furthermore, while the cerebral cortex can create and modulate cerebral electrical activity on its own, subcortical structures, particularly the thalamus, play a significant role in the creation and regulation of this activity. As mentioned, generally EEG is measured on the scalp. EEG. Electrodes, signal amplification and replication systems are crucial components.

Electrodes come in a variety of shapes and sizes (scalp-fixed, headset-fixed and hypodermic needle-fixed). EEG investigations are multichannel with two electrodes attached to each channel. Different electrodes must be positioned in standard positions on the skull’s skin. Preliminaries uses of EEG have been devoted to study electric field features [130]. Different authors measured EEG signals to predict motion intention, thus, realizing a Brain Computer Interface (BCI) to command an exoskeleton [128] for healthy or pathological subjects eventually in combination with other techniques, such as EMG or functional electrical stimulation (FES) [131].

As already seen, the EEG signal can be very different between subjects. This difference is further increased in the case where a subject has a pathology that affects the functionality of the brain, such as stroke, Parkinson’s disease, severe acquired brain injury etc. Therefore, any use of the EEG signal must be highly customized and must include a learning period. In rapidly evolving diseases, such as acute and subacute strokes, EEG features must be recalibrated at regular time intervals.

#### 4.2.5. Cameras and Optical Vision System

RGB cameras, infrared cameras, wide angle cameras and three-dimensional cameras, such as stereo vision cameras and depth cameras, are examples of video sensors that may be used to collect data. Depth cameras are made up of infrared sensors and an RGB camera, with the data received by the second camera being represented in three-dimensional space using mathematical models based on infrared ray emission and detection.

Stereovision cameras, based on the stereovision principle, often require two or more sensors to capture three-dimensional information of a subject through the triangulation process. Thermal cameras and other types of equipment can be used to assess peripheral vascular function during mobility. The only application of video cameras observed in the literature for use with exoskeletons consists of systems to validate the motion realized by these devices once worn or to generate joint motion patterns to be provided to exoskeletons by observing the natural motion of human subjects in the absence of exoskeletons.

In the work of Pan et al. [132], a six-camera infrared motion capture system was used to capture the motion of healthy and pathological subjects, which was used to realize command motion profile or to project the rehabilitation therapy. In the work by Jones et al. [133], a two-camera setup employing high-resolution, monochrome CCD cameras was used to measure exoskeleton joint angles for comparison with encoder-measured angles. Markers were attached to the exoskeleton to record movement. In the work by Chen et al. [134], the markers were covered with ultraviolet-sensitive fluorescent paint and illuminated with a UV light source.

#### 4.2.6. Encoders

The angular position or motion of a shaft or axle is converted to analog or digital output signals by a rotary encoder, also known as a shaft encoder. A shaft encoder outputs rotation angle signals in the form of pulses, which are then fed into a signal conditioning system that converts the signal to a more usable form. As with many motors and similar devices, there is often some play in the shaft between its two ends. This makes it difficult to measure the absolute position of this device using only analog inputs, such as voltage or potentiometers. This requires the use of digital inputs or software-based subsystems for measurement and feedback control. The angular position of a shaft encoder can be measured by an output test gauges that monitors the torque on an axis on a stationary point and indicates what angle corresponds to what amount of rotation in degrees.

Absolute [135] and incremental rotary encoders are the two most common variants. An absolute encoder is an angle transducer since its output indicates the current shaft position. An incremental encoder’s output offers information on the shaft’s motion, which is often processed into information, such as position, speed and distance. In exoskeletons, they are used in the proximity of a rotary motor [72] or in a rotary joint of the exoskeleton [119]. The first solution is adopted in low cost approaches and when the transmission between motor and joint is sufficiently rigid.

The second solution is adopted to accurately monitor the relative motion of two adjacent body segments around a human joint or the relative motion of two adjacent exoskeleton segments around an exoskeleton joint. In complex solutions, encoders are fused [124] with other sensors to realize a whole information acquisition system. When the structure of the exoskeleton is sufficiently rigid, encoders can be used to measure anticipatory movements and to forecast movement intention [136].

Sometimes, encoders are also adopted in elastic structures [52]. In general, mechanical, optical and magnetic are the most widely used technologies. A mechanical encoder can be rotary or linear. In most cases, applications in exoskeletons involve rotary encoders. In this type of system, there is a disk, normally metallic or otherwise rigid, with concentric rings rotating integrally with the shaft. The opening and closing of cavities produces an encoding, according to a binary code, of the relative position between a fixed reference and a mobile one. It is possible, under certain design conditions, to realize multi-turn encoders in which the position encoding is relative to a sequence of rotary movements that take place on a trajectory greater than 360 degrees.

An optical encoder comprises a shaft attached to a circular disc with one or more tracks of transparent and opaque sections that alternate. Each track has a light source and an optical sensor on opposite sides. The light sensor releases a series of pulses as the shaft rotates, interrupting the light source with the pattern on the disc. This output signal can be used with digital circuitry directly. Due to the number of output pulses, each rotation of the disc is known, and the number of output pulses per second may be translated directly to the shaft’s rotational speed.

Magnetic encoders exploit the magnetic field produced by a source located on the shaft, and, in this case, they are called on-axis, or on the hub. In the other case, they are called off-axis. Their advantage lies in the ability to operate even in disturbed contexts. In the case of potential magnetic interference, there are sensors that are properly shielded and, therefore, insensitive to environmental magnetic conditions.

#### 4.2.7. Other Sensors

A wide range of different other types of sensors are used within exoskeletons as sources of information to correct system motion in real time via a controller, to evaluate the motion characteristics of a subject wearing an exoskeleton [43,45,60], to command/modulate the realized motion or simply to identify a more or less natural motion that can be used as a set-point for the exoskeleton itself.

In the work by Crea et al. [137], capacitive sensors in orthopedic cuffs on the shanks were used to control a robotic hip orthosis. Sometimes, an alternative type of mechanical transmission used involves the use of different sensors than usual. For example, in the work by Ismail et al. [138], an infrared sensor was used as limit switch within a mechanism based on a high precision lead screw, which moved a tendon cable able to transfer the movement from the actuator to the interface between human and exoskeleton.

Sensors can be placed near the actuator that makes a standard movement, on a joint that allows fairly simple relative movements between body segments or directly on the body segments measuring the absolute movement can also be complex since this is produced by a multiplicity of actuators. Hybrid sensor network positioning is present in the literature [74]. For example, in the works by Rudd et al. [139] and by Ertas et al. [121], the authors presented low cost exoskeleton solutions exploiting a simple potentiometer integral with the driven shaft of a gearbox downstream of a simple DC motor. In the paper by Hunt et al. [140], piezo-resistive sensors were inserted in a theoretic project of a shoulder exoskeleton.

The accuracy and precision requested for this application is limited; thus, simple technologies, such as potentiometers [88,141,142], are often adopted as well as other alternative solutions, such as Hall effect sensors [143]. During walking, it can be interesting to know in which gait phase of the subject is at that moment to evaluate the naturalness of the movement and also to provide feedback to a possible control system that can make corrections. To obtain this information, insoles are often used and placed under the sole of the foot; they essentially use pressure or deformation sensors, as is the case in the work of Wang et al. [144].

Some authors designed innovative soles [42] for this application. In these exoskeletons, it is common to obtain information from the joint position and/or torque and also insole sensors [91,145] to feed the controller. When hydraulic actuators are used [90], it is possible to have information on the mechanical actions exchanged between the human subject and the exoskeleton simply by measuring the fluid pressure even in the proximity of the actuator, given the low compressibility of the fluid used for the transfer of mechanical energy inside the transmission system.

There are also hybrid solutions in the literature [89,146,147,148] that use various types of sensors even under conditions of hydraulic actions [149], and sometimes authors speak explicitly about the sensor fusion approach [126,150]. An interesting solution, even if it presents a low level of accuracy and precision, consists of measuring the electricity absorbed by a motor of an exoskeleton in terms of the current and voltage [151] in order to know the mechanical power delivered by the motor in terms of the torque and speed due to the knowledge of the electromechanical characteristics of the motor itself.

The most complex sensing system is one that allows knowledge of the movement of the distal elements [152] of a limb to provide haptic interface input for the purpose of interaction with a real, virtual or distant environment. To simplify this sensing process, some authors [115], who noted the fact that the exoskeleton is integral to the body segments and that its joints have centers of instantaneous rotation quite coincident with the relative centers between the body segments, proposed to measure the motor torques of all the actuators to combine them to, thus, obtain an estimate of all the forces acting on the exoskeleton.

A similar solution adopts a kinematic approach to identify gait phase only with joint sensors [153]. It can be interesting, for various reasons to learn about the behavior of the muscles of the subject wearing the exoskeleton. We have seen that the commonly used technique is EMG, which measures the electrical activation of muscles. An alternative technique is represented by the Force MyoGraphy (FMG) [154], which measures the change of muscle stiffness in a non-invasive way through sensor bands worn during the movement produced precisely by these changes in stiffness.

As it is possible to devise designs of underactuated exoskeletons that are compliant with the structure of the human body, similarly, some authors used flexural sensors, for example, through the use of piezo-resistive materials [112] to identify forces exchanged between the subject and the exoskeleton or by building new non-contact optical sensors [155] that measure the forces exchanged by analyzing the optical deflection of a light beam emitted from a source. Other low-cost approaches consist of developing sensorless solutions [73] that exploit classical Series Elastic Actuators (SEAs), which allow the adaptation between exoskeleton and human subject by incorporating within it passive elements, such as springs, or even active ones, for example magnetorheological SEAs [71].

In these cases, sometimes calibration is performed via external sensors only offline, such as in the work by Kim et al. [156] with an isokinetic dynamometer. An alternative solution [59] considered non-biological-based sensors, such as a Muscle Circumference Sensor (MCS) and load cells to estimate motion intention. As mentioned, the direct measure of distal body segments can be complex, but it is fundamental when rapid and accurate information must be used to interact with a virtual or real environment.

Interesting solutions can be found in the work by Ben-Tzvi et al. [157], who used force sensors on the distal parts of a glove exoskeleton to assist the subject during manipulation tasks, or in the work by de Rossi et al. [158], who adopted a tactile sensor on the distal part of the exoskeleton. A complex solution was proposed in the paper by Jones et al. [133], where the joint angles were computed from the motor shaft rotations from optical encoders integrated into each motor, and the joint torque was computed from the contact forces measured at each finger segment with a custom contact rods realized with two beams with four strain gauges.

Some authors presented innovative strain sensors, such as Tjahyono et al. [159], who proposed a new polypyrrole strain sensor attached to artificial pneumatic muscle actuators. An intermediate approach is to place the sensors after part of the drive train; this allows a tradeoff between a compact design and reducing errors in the drive train. This is the case in an example in the literature [160], where a position sensor is allocated after a long yielding transmission, but before the last part of the transmission, in an exoskeleton glove. A similar solution was adopted in the work by Wang et al. [50] for potentiometer and strain gauge sensors located after a flexible shaft and before the last part of the transmission, or in a similar way in the paper by Agarwal et al. [61] with magneto-resistive angle sensors, or in the work by Aubin et al. [62] with embedded encoders and bend sensors.

In conclusion, when the transmission is sufficiently stiff, the kinematics can be measured before the transmission; whereas, the interacting force is typically measured near the distal part of the exoskeleton in particular when there is an interaction with the environment [161]. A time-demanding operation for exoskeletons is mounting, i.e., the wearing operation; thus, sometimes semiautomatic techniques adopting sensors are implemented to accelerate this phase, as in the work by Nef et al. [162], which adopts laser diodes to define the correct position of a subject using an upper limb exoskeleton.

## 5. Conclusions

This paper aimed at investigating the current state of the art on the subject of sensors and actuation technologies in exoskeletons in a comprehensive way, i.e., with a review not focused on devices with a definite purpose or devoted to a specific body district. The analysis was performed on 215 documents, journal articles and reviews with no year limitations. The study was performed with a dual-level investigation:

i. The prospective review, which generated the classification of the documents by purpose, focus, anatomical district, device type and design solutions and allowed the observational study of the literature evolution over time by purpose and by focus.
ii. The analytical review, which generated for the power source, actuation technology and sensors, a mapping of the documents, by actuators, sensor type and innovative actuation and sensing methods.

At the analytical review level, cross analyses among different aspects were developed for a more in-depth investigation of the correlations between the various topics. Table 2 and Table 3, presented the main quantitative findings of perspective and analytical reviews, respectively. Within the subparagraphs of the analytical review, a detailed qualitative discussion of the main solutions reported in the literature for the exoskeleton actuation and sensing technologies was developed, in an attempt to extrapolate the most relevant and innovative aspects from the analyzed articles.

Those evaluations aimed at providing the reader with a handy support for the interpretation of the best practices currently adopted according to the analyzed literature. Considering the significant in-depth level of the analysis, we expect that this work could provide engineers, physiotherapists and exoskeleton developers with useful ideas and information for their activities. Furthermore, the developed work could represent a map of the treated themes that can have a valuable utility for consensus initiatives (such as consensus conferences) and certification. Nevertheless, the proposed taxonomies were designed to capture at best the peculiarities of the current dataset according to the purpose of the review; alternative taxonomies could be more suitable to emphasize different features of the same dataset.

The research and development work related to exoskeletons from about 2008 to date has been enormous and has addressed a huge variety of different themes and issues. Given the complexity of these devices, also due to the safety requirements for the close interaction with humans, there are many design challenges still unresolved, which have considerable room for improvement, or for which future innovations will be decisive. Actuator dimensions, system portability, battery time-span and user comfort are a few examples.

In this context, a review of the same literature with a different focus could certainly provide useful input. Referring to the scheme in Figure 1, interesting aspects could emerge from literature reviews on the topics of control and related algorithms [85,163,164], specifically regarding the mechanical structure [11,165,166] or the HMI interfaces [167,168].

## Figures and Tables

**Table 1:** The main reviewed publications on the exoskeleton theme.

Ref.	First Author	Year	Purpose	Anatomical District	Main Topics
[27]	Hussain F.	2021	Assistance	LLE	Materials, actuation, and manufacturing methods
[17]	Sanjuan J.D.	2020	Rehabilitation	ULE	Design review of cable driven exoskeletons
[1]	Agarwal P.	2019	Rehabilitation, assistance, performance augmentation	All	Design, Actuation, Sensing, Materials, Control, Case studies
[28]	Shi D.	2019	Rehabilitation	LLE	Human gait analysis, Design, Actuation, Control
[29]	Sanchez-Villamañan M.	2019	Rehabilitation and assistance	LLE	Mechanical design principles in compliant LLE
[9]	Al-Shuka H.F.N.	2019	Power augmentation exoskeletons	LLE	Biomechanics, actuation, control
[23]	Rehmat N.	2018	Rehabilitation	ULE	Mechanical design, control, clinic trials
[25]	Manna S.K.	2018	Rehabilitation	ULE	Actuation systems
[30]	Zhang X.	2017	Rehabilitation	LLE	Overview of recent representative robots,actuation, control
[31]	Louie D.R.	2016	Rehabilitation	LLE	Clinical trials of robotic exoskeletons for gait rehabilitation in adults post-stroke
[32]	Chang S.R.	2015	Rehabilitation	LLE	Overview of commercial devices
[24]	Blank A.A.	2014	Rehabilitation	ULE	Patient Engagement in Therapy
[26]	Maciejasz P.	2014	Rehabilitation	ULE	Overview devices, type of assistance, mechanical design, actuation control, clinical studies
[33]	Heo P.	2012	Rehabilitation and assistance	Hand	Biomechanics, overview devices, actuation, intention sensing methods

**Table 2:** Synopsis of the main findings of the prospective review.

Topic	Findings
Time	(F1) The beginning of scientific production on this theme can be placed around 2006, initially mainly with conference works.
	(F2) Starting from 2011, a significant growth trend begins, both for resident jobs and for conference jobs, and a similar growth trend is observed for these two different types of contributions.
Purpose	(F3) Journal publications mainly concern exoskeletons intended for rehabilitation or medical applications, followed by those for assistive devices for people with permanent disabilities.
	(F4) Studies on exoskeletons for rehabilitation (or more generally for medical applications) and assistive devices cover the 58% and the 38%, respectively.
	(F5) Publications on exoskeletons to increase performance are about 14% of the total, while for haptic systems this is on the order of a dozen.
	(F6) A growth trend in the scientific production of articles for medical applications occurs since 2013 and around 2019 the growth rate of related papers increases.
Focus	(F7) About 52% of the revised publications have the actuation system as the main focus and 30% concern sensors. This trend could be expected considering that actuator dimensions, weight, consumption are still critical elements of the overall design of exoskeletons.
	(F8) 2016 marks the beginning of a greater research in actuation systems, and the research on assistive devices began growing in 2016 as well. These trends are most likely correlated, as portable assistive devices require very energy-efficient, very compact, less heavy and more dynamically performing actuation systems.
Anatomical District	(F9) The lower limb exoskeletons are the most investigated (57%) and to the knee joint in particular (78% of lower limb related works).
	(F10) Among the devices for the upper limbs, the most frequent ones in publications are devoted to the elbow (74%).
	(F11) Among the hand exoskeletons, the devices that treat more fingers are the most investigated ones (71%).
Device Type	(F12) Actuated exoskeletons cover almost the full amount of devices, and active-assisted rehabilitation is the most frequent.
Design Solutions	(F13) Portability of the exoskeletons is a technical characteristic analyzed in about the 20% of the documents.
	(F14) 42% of the design choices for motion transmission fell on cables. Using the cable transmission system results in a significant reduction in the exoskeleton’s weight and in the required torque at the joint level. This solution is particularly useful in fixed devices to lighten the device from the actuators that remain on the ground. Furthermore, when SEA electric actuators are used, Bowden cables are typically used, which contribute to introducing an elastic component into the system.
	(F15) Velcro and latches are the common adopted solutions for the joining between human and exoskeleton.
	(F16) A lower number of DoF is preferred for both active and passive DoF. These trends fit well with the tendency towards simple and compact design strategies
Anatomical District vs. Purpose	(F17) Rehabilitation/medical exoskeletons are predominant for ULE, hand and forearm exoskeletons, while assistive devices are predominant for LLE.
	(F18) The predominance of LLE is confirmed regardless of the purpose of the device, and, in assistive devices, it has the highest percentage.
	(F19) The power/performance augmenting exoskeletons were developed for all the anatomical districts with small percentages.

**Table 3:** Synopsis of the main findings of the analytical review.

Topic	Findings
Actuation Technology/ Power source	(F20) Electric power source is undoubtedly the most diffused.
	(F21) Pneumatics and hydraulics are much less used than the electric solution, and, in particular, hydraulics is the least used of all.
Actuation Technology/ Actuator types	(F22) Brushed and brushless DC motors are the most used electric actuators.
	(F23) The main advantages of brushless vs. brushed motors are the greater reliability, due to the lack of brushes and the better dynamic performance allowed by a lower rotor inertia and the higher power-to-weight ratio.
	(F24) In recent decades, SEAs have been increasingly used. They allow a smooth force transmission, accurate force control, lower output impedance, shock tolerance, energy efficiency and back-drivability. Therefore, they allow a safe human–robot physical interaction.
	(F25) Other very promising actuators for exoskeletons belong to the class of Variable Stiffness Actuators (VSAs).
	(F26) PAM are the most diffused actuators for pneumatically actuated exoskeletons. Soft actuators are used in a limited number of cases and there are applications for the lower limbs and for the hand.
	(F27) Some researchers proposed a hybrid electric-pneumatic actuation, in which the pneumatic drive takes care of the initial reaction of the force, and the electric drive complements the pneumatic drive.
Sensors	(F28) Dynamic sensors are predominant, with a significant number or force and torque sensors. Encoders are also quite diffused.
	(F29) Cameras and Optical Vision System are mainly used to validate the motion realized by these devices once worn or to generate joint motion patterns to be provided to exoskeletons by observing the natural motion of human subjects in the absence of exoskeletons.
	(F30) A wide range of particular sensors are used in exoskeletons (e.g., infrared, capacitive, FMGs, MCSs and laser diodes sensors)
Anatomical District vs. Power Source	(F31) The predominance of electric actuation is independent on the anatomical districts to which the exoskeleton is aimed at.
	(F32) Pneumatic and hydraulic actuation are mainly adopted in lower limb exoskeletons.
	(F33) Successful implementations of hydraulic actuators are mainly in the LLEs, for which the load capability is one of the most important requirements.
Anatomical District vs. Actuator types	(F34) Brushed DC motors are predominant in ULE and hand exoskeletons.
	(F35) In LLE brushless motors are the most used actuators.
	(F36) Particular actuators, as SMA, EAP or magneto-rheological fluids are very little used and never for LLE.
	(F37) Hydraulics is not used in revised works with hand exoskeletons.
	(F38) SEAs are similarly diffused in ULE, LLE and hand exoskeletons.
	(F39) PAMs are mainly used in exoskeletons for rehabilitation
	(F40) Hydraulic actuation is mainly used in assistive devices or for power/performance augmentation.
	(F41) LLE is the predominant anatomical district for all the actuator types (due to the greater diffusion of these exoskeletons).
Purpose vs. Power Source	(F42) Electric actuation is the predominant choice regardless of the exoskeleton purpose.
	(F43) Pneumatics is equally present in EDs for medical applications and power/performance augmentation with a limited percentage.
	(F44) Hydraulics has a significant role in power/performance augmentation EDs, due to the need to move high loads.
Purpose vs. Actuator types	(F45) For the most adopted electric actuators (DC, brushless and SEAs), quite similar weights of medical and assistive purposes is observed.
	(F46) Brushed DC is the most frequent choice for all the ED purposes.
	(F47) VSAs, torque and linear motors, which are overall little used, are mainly diffused in medical or for rehabilitation prototypal devices.
Anatomical District vs. Sensors	(F48) For ULEs, LLEs and HEDs, a significant variety of sensors is used, and for all, there is not one sensor type that is significantly more used than the others.
	(F49) For ULEs, a small prevalence of encoders emerges.
	(F50) For LLEs, a small prevalence of force sensors emerges.
	(F51) For HEDs, force sensors and encoders are the two main classes.
Sensors vs. Power Source	(F52) With electric actuation, encoder and force sensors are the most used sensors; EMGs, IMUs and torque sensors follow with quite lower percentages.
	(F53) With pneumatics, pressure sensors and force sensors are equally frequent and are the most used.
	(F54) With hydraulics, pressure sensors are dominant with 27%, while force sensors, torque sensors, IMUs and encoders are equally present (18%).
Sensors vs. Actuator types	(F55) Encoders are an important and almost equal presence with brushed DC motors, SEAs and brushless motors.
	(F56) Force sensors are widely used with SEAs and VSAs and with fluidic actuators.
	(F57) Pressure sensors are highly used with soft actuators, PAMs and hydraulic cylinders.

**Figure A1:**
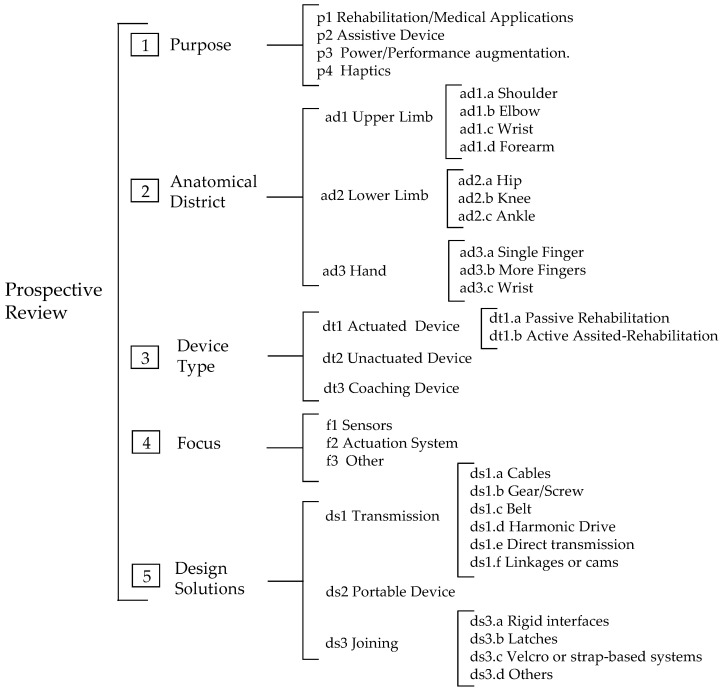
Adopted taxonomy for the review.

**Figure A2:**
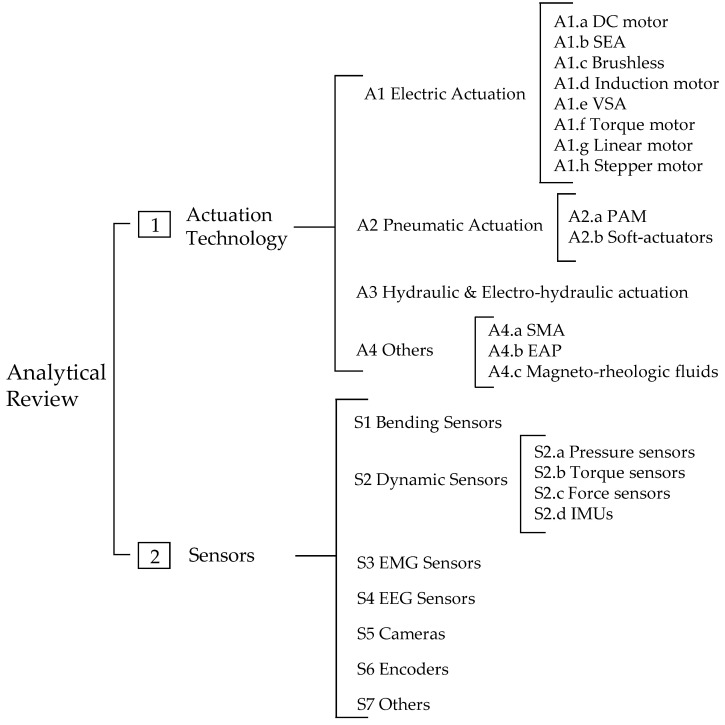
Adopted taxonomy for the analytical review.

## Abbreviations

The following abbreviations are used in this manuscript:

ADLs	Activities of Daily Living
ALS	Amyotrophic Lateral Sclerosis
ARES	Adjustable Rigidity and Embedded Force Sensors
BCI	Brain Computer Interface
CP	Cerebral Palsy
CPU	Central Processing Unit
DC	Direct Current
DoF	Degrees of Freedom
EAP	ElectroActive Polymer
EAsoftM	Exoskeleton Actuated by the soft Modules
ED	Exoskeleton Device
EEGs	ElectroEncephalogGraphy signals
EMGs	ElectroMyoGraphy signals
eSEA	Elastamor-Based Series Elastic Actuator
ESGP	Echo State Gaussian Process
FES	Functional Electrical Stimulation
FMG	Force MyoGraphy
FSRs	Force-Sensitive Resistors
HED	Hand Exoskeletal Device
HMI	Human-Machine Interface
IMU	Inertial Measurement Unit
LLE	Lower Limbs Exoskeleton
MMG	MechanoMyoGraphy signals
MR	MagnetoRheological
MRSEA	Magneto-Rheological Series Elastic Actuators
NMPC	Nonlinear Model Predictive Control
PAM	Pneumatic Artificial Muscles
PEA	Parallel Elastic Actuators
PPAM	Plated PAM
PREHydra	Passive Return Electro-Hydrostatic actuator
PSMC	Proxy-based Sliding Mode Control
QDD	Quasi-Direct Drive
SCI	Spinal Cord Injuries
SEA	Series Elastic Actuators
SEAC	Series of Elastic Actuators with Clutch
SMA	Shape Memory Alloys
ULE	Upper Limbs Exoskeletons
VIAs	Variable Impedance Actuators
VSA	Variable Stiffness Actuators

## Appendix A. Description of Classification Approach and Tables

This Appendix contains the tables resulting from the classification performed following the criteria and parameters explained in Section 2.2. For the classification, a pragmatic method, which proved to be very effective, was followed. The classification approach is described below, whereas Figure A1 and Figure A1 provide a synthetic overview of the taxonomy adopted for prospective and analytical review.

An Excel sheet with the documents in the lines and complete taxonomy classes and subclasses in the columns was preliminary produced and subsequently filled while reading the full paper. From this worksheet, four tables were extracted: the complete classification map and the cross-analysis sub-tables. Starting from the complete classification, the filter data Excel function was used to perform cross analysis and to fill the evaluated sub-tables reported in the following, based on the taxonomy of the analytical review.

Table A1 contains the cross analysis of the paper distribution of exoskeleton purpose vs. anatomical district. Table A2 contains the cross analysis of the paper distribution of power source vs. anatomical district. Table A3 contains the cross analysis of the paper distribution of power source vs. exoskeleton purpose. Table A4 contains the cross analysis of the paper distribution of sensors vs. anatomical district. Table A5 contains the cross analysis of the paper distribution of sensors vs. actuators types. Finally, Table A6 describes the results of the classification of all the documents included in the review according to the taxonomy of the review.

**Table A1:** Cross analysis of the paper distribution of exoskeleton purpose vs. anatomical district.

	ad1. Upper Limb	ad2. Lower Limb	ad3. Hand	ad4. Forearm
p1. Rehabilitation/Medical applications	[81] ${}^{1}$; [82] ${}^{1}$; [106]; [118] ${}^{2}$; [169] ${}^{1}$; [100]; [170] ${}^{1}$; [111] ${}^{1}$; [171] ${}^{1}$; [172] ${}^{3}$; [99] ${}^{1}$; [173] ${}^{1}$; [86] ${}^{1}$; [115]; [119] ${}^{1}$; [25]; [73]; [103] ${}^{1}$; [174] ${}^{1}$; [58] ${}^{1}$; [131]; [47] ${}^{1}$; [152]; [134]; [175]; [141]; [162] ${}^{1}$; [176]; [150] ${}^{1}$; [148] ${}^{1}$; [123] ${}^{1}$;	[77] ${}^{1}$; [109] ${}^{1}$; [177]; [116]; [178] ${}^{1}$; [179]; [180] ${}^{1}$; [94] ${}^{1}$; [92]; [181] ${}^{1}$; [182] ${}^{4}$; [183] ${}^{1}$; [184] ${}^{1}$; [185] ${}^{1}$; [186] ${}^{1}$; [187]; [188]; [97] ${}^{1}$; [189] ${}^{1}$; [190] ${}^{6}$; [191] ${}^{6}$; [114]; [192] ${}^{1}$; [70] ${}^{1}$; [108] ${}^{1}$; [193]; [194] ${}^{4}$; [195] ${}^{1}$; [196] ${}^{1}$; [93]; [132] ${}^{1}$; [197] ${}^{1}$; [67]; [198] ${}^{7}$; [199] ${}^{1}$; [76]; [200] ${}^{1}$; [36] ${}^{1}$; [201]; [202]; [71] ${}^{1}$; [84]; [39] ${}^{1}$; [203] ${}^{1}$; [204] ${}^{7}$; [41] ${}^{1}$; [43] ${}^{1}$; [105]; [101]; [158]; [205] ${}^{6}$; [46] ${}^{1,8}$; [98]; [156]; [63]; [129]; [176]; [150] ${}^{1}$;	[206] ${}^{1}$; [207] ${}^{1}$; [51] ${}^{1}$; [106]; [113]; [208]; [209] ${}^{1}$; [210] ${}^{1}$; [139] ${}^{1}$; [52] ${}^{1}$; [1] ${}^{1}$; [110] ${}^{1}$; [211] ${}^{1}$; [155]; [49]; [157] ${}^{1}$; [160] ${}^{1}$; [50]; [60]; [212]; [62]; [121]; [133]; [159]; [213]; [129];	[57] ${}^{1}$; [147] ${}^{1}$;
p2. Assistive device	[214] ${}^{1}$; [215]; [100]; [111] ${}^{1}$; [68] ${}^{1}$; [216] ${}^{1}$; [217]; [138] ${}^{1}$; [154] ${}^{1}$; [140] ${}^{1}$; [218]; [103] ${}^{1}$; [150] ${}^{1}$;	[27]; [219] ${}^{1}$; [177]; [220]; [69] ${}^{1}$; [179]; [79] ${}^{1}$; [117] ${}^{1}$; [184] ${}^{1}$; [221] ${}^{1}$; [186] ${}^{1}$; [222] ${}^{1}$; [223] ${}^{1}$; [64] ${}^{1}$; [224] ${}^{1}$; [65] ${}^{1}$; [87] ${}^{1}$; [225] ${}^{9}$; [226] ${}^{1}$; [70] ${}^{1}$; [108] ${}^{1}$; [227] ${}^{1}$; [144] ${}^{1}$; [228] ${}^{1}$; [102] ${}^{1}$; [128] ${}^{1}$; [124] ${}^{1}$; [137] ${}^{1}$; [75]; [136]; [90] ${}^{1}$; [151] ${}^{1}$; [72] ${}^{1}$; [229] ${}^{1}$; [197] ${}^{1}$; [67]; [140] ${}^{1}$; [89]; [230] ${}^{1}$; [76]; [112] ${}^{1}$; [200] ${}^{1}$; [37] ${}^{1}$; [231] ${}^{1}$; [201]; [74] ${}^{1}$; [39] ${}^{1}$; [232] ${}^{1}$; [105]; [205] ${}^{6}$; [78]; [233]; [145]; [98]; [143]; [234]; [129]; [235];	[107] ${}^{1}$; [236] ${}^{1}$; [237]; [224] ${}^{1}$; [52] ${}^{1}$; [110] ${}^{1}$; [155]; [213];	
p3. Power/Performance augmentation	[106]; [238]; [239] ${}^{1}$; [240] ${}^{1}$; [174] ${}^{1}$; [142] ${}^{1}$; [148] ${}^{1}$;	[117] ${}^{1}$; [83]; [241] ${}^{1}$; [135] ${}^{1}$; [9]; [200] ${}^{1}$; [74] ${}^{1}$; [242] ${}^{1}$; [84]; [146] ${}^{1}$; [88] ${}^{1}$; [42]; [101]; [44] ${}^{1,5}$; [98]; [125]; [122]; [235];	[106]; [56] ${}^{1}$; [45] ${}^{1}$; [129];	[126] ${}^{1}$;
p4. Haptics	[243] ${}^{1}$; [115]; [244] ${}^{1}$;	[245] ${}^{1}$; [84]; [234];	[157] ${}^{1}$;	

**Notes**: ^1^ Custom made exoskeleton. ^2^ Commercial exoskeleton EXO-UL8. ^3^ Commercial exoskeleton ARMEO-POWER. ^4^ Commercial exoskeleton Ekso LOKOMAT or EksoGT. ^5^ Commercial exoskeleton EXOwheel. ^6^ Commercial exoskeleton REX. ^7^ Commercial exoskeleton Rewalk. ^8^ Commercial knee exoskeleton EXO-H2, USW-UFES’s Smart Walker. ^9^ Commercial exoskeleton AIDER.

**Table A2:** Cross analysis of the paper distribution of power source vs. anatomical district (DC: Direct Current; SEA: Series Elastic Actuator; VSA: Variable Stiffness Actuator; PAM: Pneumatic Artificial Muscle; SMA: Shape Memory Alloy; and EAP: Electro-Active Polymer).

Actuation Type	ad1. Upper Limb	ad2. Lower Limb	ad3. Hand	ad4. Forearm	ad5. Trunk
A1. Electric Actuation					
A1.a DC	[106]; [118]; [170]; [217]; [138]; [119]; [154]; [25]; [59]; [131]; [141]; [244];	[219]; [79]; [180]; [182]; [184]; [221]; [186]; [223]; [194]; [241]; [124]; [137]; [136]; [227]; [151] ${}^{1}$; [240] ${}^{2}$; [89]; [200]; [232]; [101]; [78]; [233]; [145]; [143]; [150];	[206]; [51]; [106]; [107]; [236]; [237] ${}^{3}$; [209]; [210]; [139]; [52] ${}^{4}$; [157]; [160]; [61]; [53]; [121]; [133]; [213];	[126];	
A1.b SEA	[81]; [82] ${}^{4}$; [69]; [86]; [119]; [73];	[178]; [220]; [92]; [188]; [70]; [193]; [241]; [137]; [72]; [115] ${}^{5}$; [231]; [71]; [74]; [84] ${}^{4}$; [39]; [203]; [63];	[207]; [107]; [52] ${}^{4}$; [203];		
A1.c brushless	[69]; [68]; [243]; [115]; [140]; [59]; [131]; [244];	[109]; [177]; [179]; [79]; [181]; [183]; [186]; [64] ${}^{6}$; [65]; [87]; [114]; [225]; [83] ${}^{7}$; [228]; [195]; [246]; [196]; [75]; [93]; [132]; [197]; [66]; [140]; [230]; [231]; [71]; [242]; [88]; [153]; [41]; [43];			[54];
A1.d induction		[185];			
A1.e VSA		[77]; [70]; [76]; [202]; [146]; [39];			
A1.f torque		[76]; [112]; [105]; [78]; [233]; [143]; [176];	[50]; [62]; [133];		
A1.g linear		[189]; [156]	[155]; [160]; [45];		
A1.h stepper		[199];			
A1.i others	[172]; [218]; [58] ${}^{8}$; [47]; [175]; [142]; [162];	[27]; [113]; [222]; [191]; [135]; [128]; [34]; [35]; [204]; [122]; [158]; [235];	[110];		[80];
A2. Pneumatic Actuation					
A2.a PAM	[171]; [99]; [134]; [148];	[94]; [245]; [111] ${}^{9}$; [97]; [96]; [108]; [37]; [247]; [101]; [98] ${}^{10}$; [125];	[212]; [159];	[147];	
A2.b soft-actuators	[103];	[224]; [226]; [102]; [234];	[224]; [56]; [60];		
A3. Hydraulic Actuation					
A3.a cylinders	[239]; [25]; [89];	[219]; [248]; [92]; [117]; [90]; [240] ${}^{2}$; [249]; [91];			
A4. Others					
A4.a SMA	[218]; [58] ${}^{8}$;		[110];		
A4.b EAP	[25];				
A4.c magneto-rehologic fluids	[214]; [215];				

**Notes**: ^1^ Sensor-less LLE powered by an electric Direct Current (DC) motor, and the same motor is used to detect motion via monitoring the voltage and the current variation. ^2^ LLE for power amplification that perceives intended human motion via human-exoskeleton interaction signals measured by torque sensors, estimates human gait trajectories and walking phase is identified by the threshold approach using ground reaction force. ^3^ Summarize existing approaches of remote actuation systems for wearable assistive technology. ^4^ SEAs with Bowden Cables. ^5^ Methodology, based on a graphical tool, for the evaluation of different actuator choices resulting from the combination of different motors, reduction gears and parallel stiffness profile. ^6^ Tests of the feasibility of an elastomer-based series elastic actuator to provide torque sensing as a haptic interface for soft, comfortable HRI in exoskeletons. ^7^ SEA force controller by measuring elastic displacements of spring elements at hip joints. ^8^ Medical rehabilitation exoskeleton for the elbow with one degree of freedom for flexion-extension, actuated with shape memory alloy wire-based actuators. ^9^ PAMs with Bowden Cables. ^10^ Pleated pneumatic artificial muscles in a novel actuator system design with high torque range and large range of motion.

**Table A3:** Cross analysis of the paper distribution of power source vs. exoskeleton purpose.

Actuation Type	p1. Rehabilitation/Medical applications	p2. Assistive Device	p3. Power/Performance Augmentation	p4. Haptic
A1. Electric Actuation				
A1.a DC	[206]; [51]; [106]; [180]; [118]; [209]; [182]; [184]; [186]; [210]; [193]; [194]; [139]; [52]; [200]; [119]; [67]; [25]; [157] ${}^{1}$; [160]; [61]; [101]; [131]; [121]; [133]; [213]; [141]; [150]; [61];	[219]; [107] ${}^{2}$; [236]; [237]; [184]; [221]; [186]; [223]; [217]; [124]; [137]; [138]; [136]; [52]; [227]; [151]; [154]; [67]; [89]; [200]; [232]; [78] ${}^{3}$; [233]; [145]; [143]; [213]; [150];	[106]; [241]; [240]; [200]; [101]; [126];	[157] ${}^{1}$; [61]; [53]; [244];
A1.b SEA	[207]; [178]; [82]; [86]; [92]; [188]; [70]; [52]; [119]; [73]; [71]; [84]; [39]; [49]; [203]; [63]; [82];	[81]; [220]; [69]; [107] ${}^{2}$; [70]; [137]; [52]; [72] ${}^{4}$; [231]; [74]; [39];	[241]; [74]; [242]; [84];	[84];
A1.c brushless	[109]; [177]; [179]; [181]; [183]; [187]; [195]; [196]; [93]; [132]; [115]; [197]; [201]; [153]; [41]; [43];	[177]; [69]; [179]; [79]; [68]; [64]; [225]; [228]; [75] ${}^{5}$; [197]; [140]; [54]; [230]; [201]; [153];	[83]; [88];	[243]; [115];
A1.d induction	[185] ${}^{6}$;			
A1.e VSA	[77]; [76]; [202]; [39];	[76]; [39];	[146];	[76];
A1.f torque	[76]; [50]; [105]; [62]; [152]; [176]; [123];	[76]; [112]; [105];	[127];	[76];
A1.g linear	[189]; [55]; [155]; [174] ${}^{7}$; [156];	[155];	[174]; [45];	
A1.h stepper	[199];			
A1.i others	[169]; [172]; [113]; [100]; [191]; [110]; [57]; [58]; [204]; [47]; [158]; [175]; [162]; [129];	[27]; [100]; [222]; [128]; [229]; [110]; [218]; [129]; [235];	[135]; [80]; [44] ${}^{8}$; [122]; [142]; [235];	[245];
A2. Pneumatic Actuation				
A2.a PAM	[94]; [111]; [97]; [171]; [108]; [96]; [99]; [212]; [98]; [159]; [134]; [147]; [148]; [147];	[111]; [108]; [37]; [98];	[98]; [125]; [148];	
A2.b soft-actuators	[103]; [60];	[224]; [226]; [102]; [103];	[56];	
A2.c others		[27];		
A3. Hydraulic Actuation				
A3.a cylinders	[92];	[27]; [219]; [117]; [90]; [89]; [234];	[117]; [239]; [240]; [88];	[234]; [149];
A4. Others				
A4.a SMA	[110]; [57]; [58];	[110]; [218];		
A4.b EAP	[25];			
A4.c magneto-rehologic fluids	[71];	[214]; [215];		

**Notes**: ^1^ Sensing and force-feedback hand exoskeleton, which collects kinematic and force information from the human hand and playbacks the motion to assist in c hand grasping movements. ^2^ ELab tenoexo HED, which combines features of rigid link structures and soft mechanisms and allows natural grasp adaptation in an extremely lightweight and sleek design. ^3^ VSA in which the compliant elements simultaneously allow measuring of the torque exerted by the joint. ^4^ Control strategy for gravity compensation based on modeling of the full exoskeleton dynamics and of the contacts with the environment. ^5^ Development a series of elastic actuator with a mechanical clutch that automatically disengages the transmission when needed. ^6^ Systematic method to size the motor-transmission unit by taking into account the motor’s characteristics, transmission inertia, efficiency and cost of the system. ^7^ Five DoF low inertia shoulder exoskeleton, with a 3DoF spherical parallel manipulator and a 2DoF passive slip interface used to couple the user upper arm to the spherical parallel manipulator. ^8^ Estimation of the user muscular efforts using joint torque sensor.

**Table A4:** Cross analysis of the paper distribution of sensors vs. anatomical district.

Sensors	ad1. Upper Limb	ad2. Lower Limb	ad3. Hand	ad4. Forearm	ad5. Trunk
S1. Bending Sensors	[106];	[108] ${}^{1}$;	[106]; [79]; [56]; [62];		
S2. Dynamic Sensors					
S2.a pressure	[103];	[109]; [245]; [186]; [97]; [108]; [226]; [102]; [137]; [90]; [197]; [240]; [74]; [42]; [43]; [105] ${}^{2}$; [205]; [98] ${}^{3}$; [158];	[56]; [147];	[126] ${}^{4}$;	
S2.b torque	[118]; [239] ${}^{5}$; [115]; [175]; [141];	[92]; [223]; [65]; [87]; [114]; [197]; [240]; [105]; [44]; [78];	[80]; [50] ${}^{6}$; [45]; [147];		[54] ${}^{7}$;
S2.c force	[239]; [218]; [235]; [176] ${}^{8}$; [150];	[111]; [68]; [92]; [117]; [70]; [194]; [97]; [229]; [197]; [230]; [112]; [200]; [249]; [36]; [231]; [71]; [74]; [242]; [247]; [39]; [232]; [204]; [42]; [91]; [205]; [98]; [125]; [234]; [158];	[113] ${}^{9}$; [110]; [155]; [56]; [157]; [60]; [53]; [213];	[57];	
S2.d IMU		[116]; [69]; [180]; [181]; [184] ${}^{10}$; [221]; [186]; [187]; [102]; [226]; [108]; [128]; [75]; [72]; [197]; [34]; [35]; [200]; [71]; [43];			[54];
S3. EMG Sensors		[68]; [216]; [217]; [119]; [218]; [47]; [150];	[182]; [189]; [192] ${}^{11}$; [83]; [128]; [124]; [120]; [37]; [247]; [232]; [101]; [46]; [125]; [122]; [234]; [63]; [129];	[206]; [51] ${}^{12}$; [160]; [121]; [129];	[126];
S4. EEG Sensors	[131] ${}^{13}$;	[177]; [179]; [222]; [190]; [191]; [173]; [196]; [128]; [46];	[212];		
S5. Cameras	[103]; [40]; [174]; [134];	[132] ${}^{14}$; [74]; [146]; [247]; [153];	[49]; [133];		
S6. Encoders	[115]; [119]; [140]; [218]; [73]; [40]; [174]; [131]; [141] ${}^{15}$; [235];	[114]; [246]; [135]; [124]; [136]; [72]; [229]; [197]; [55]; [140]; [240]; [34]; [35]; [76]; [71]; [242]; [84]; [39]; [88]; [41]; [105]; [98]; [125];	[52]; [155]; [61]; [53]; [45]; [62]; [121]; [147];	[126];	[54];
S7. Other Sensors	[238]; [111]; [216]; [99]; [138]; [115] ${}^{16}$; [59]; [162]; [150];	[181]; [188]; [224]; [228]; [196]; [128]; [137]; [144]; [90]; [151]; [42]; [91]; [145];	[236]; [113]; [224]; [209]; [139]; [49]; [157]; [160]; [50]; [60];		

**Notes**: ^1^ Soft-rigid pneumatic exoskeleton for lower limb, with three soft hinges (to drive the hip, knee and ankle joints) and four rigid links (aligned with the waist, thigh, shank, and foot.) ^2^ The authors developed an in-sole foot sensor for a knee exoskeleton. ^3^ Use of pleated pneumatic artificial muscles and each PPAM is equipped with a gauge pressure sensor. ^4^ Sensor fusion with the skin surface EMG signals and the generated wrist force signal in an exoskeletal robot for human elbow motion support. ^5^ Six-axis force/torque sensor installed between the exoskeleton and the end effector in an application for load compensation. ^6^ Joint angle and torque measurements are used for feedback control in a exoskeleton for thumb motion. ^7^ An absolute magnetic encoder measures angles of hip-flexion/extension, and an IMU is installed on the back part to estimate orientation of a wearer’s torso. ^8^ Spring are used as a compliant and relatively low-cost force sensor. ^9^ Design of an elastic sensing unit for finger spasm force measurement. ^10^ Fuzzy-logic-based algorithm for the locomotion mode and locomotion transition recognition with an active pelvic orthosis. ^11^ EMG-driven speed-control of a LLE to provide better interaction between a user and the system; the gait cycle duration (was extracted from sEMG signals using the autocorrelation algorithm and Bayesian fusion algorithm. ^12^ An HED is controlled according to the motion intention of the subjects through EMGs; five classifiers including support vector machine (SVM), K-near neighbour (KNN), decision tree (DT), multilayer perceptron (MLP) and multichannel convolutional neural networks (multichannel CNN) were compared for the offline classification. ^13^ Brain–computer interface-controlled exoskeleton/functional electrical stimulation training device with proprioceptive feedback. ^14^ Gait pattern at comfortable walking speed obtained by utilizing a six-camera infrared motion capture system. ^15^ Validation of the suitability of a novel rotational hydroelastic actuator, which is a combination of a symmetric torsion spring, a rotational hydraulic actuator, and high-precision quadrature encoders. ^16^ Elastomer based SEA which acts as a soft interface and sense human/robot interface torque.

**Table A5:** Cross analysis of the paper distribution of sensors vs. actuator types.

Actuation Type	S1. Bend	S2. Dynamic Sensors				S3. EMG	S4. EEG	S6. Encoders
		S2.a Pressure	S2.b Torque	S2.c Force	S2.d IMU			
A1. Electric								
A1.a DC	[106]; [107];	[186]; [137]; [240]; [89]; [126];	[118]; [223]; [240]; [78]; [126];	[194]; [200]; [232]; [157]; [53]; [213]; [244]; [150]; [127]; [123];	[180]; [184]; [221]; [186]; [200];	[206]; [51]; [182]; [217]; [124]; [119]; [232]; [160]; [101]; [121]; [150]; [127]; [123];	[131];	[124]; [136]; [52]; [119]; [154]; [89]; [61]; [53]; [131]; [121]; [244]; [126]; [127]; [123];
A1.b SEA	[107];	[137]; [74];	[141];	[70]; [231]; [71] ${}^{1}$; [74]; [39];	[69];	[63];		[52]; [84]; [39]; [141];
A1.c brushless		[109]; [197]; [43] ${}^{2}$;	[65]; [87]; [114]; [115]; [197];	[68]; [197]; [230]; [231]; [242]; [38]; [244];	[181]; [187]; [75]; [72]; [197]; [54]; [71] ${}^{1}$; [43] ${}^{2}$;	[68]; [83]; [38];	[177]; [179]; [68]; [196]; [131];	[114]; [246]; [72]; [115]; [197]; [71] ${}^{1}$; [242]; [88]; [41]; [43] ${}^{2}$; [131]; [244];
A1.d induction								
A1.e VSA				[70]; [76]; [39];				[39]; [88];
A1.f torque	[62];	[105];	[50]; [105]; [78];	[112]; [176];	[152];			[62];
A1.g linear			[45];	[155] ${}^{3}$;		[189]; [160];		[55]; [174] ${}^{1}$;
A1.h stepper	[107];				[199];			
A2. Pneumatic								
A2.a PAM	[108];	[245]; [97] ${}^{4}$; [108]; [147];	[147]; [148];	[97] ${}^{4}$; [111]; [247]; [98]; [125];	[108];	[37]; [101]; [125];	[212];	[98]; [125]; [147];
A2.b soft-actuators	[56];	[226]; [102]; [56]; [103] ${}^{5}$;		[56]; [60]; [234];	[226]; [102];	[234];		[88]; [234];
A3. Hydraulic								
A3.a cylinders		[240]; [89]; [149];	[239]; [240];	[239]; [91] ${}^{6}$;	[34]; [35];			[89]; [149];

**Notes**: ^1^ VICON vision system used for experimental setup. ^2^ Force-sensitive resistors (FSRs) in the user’s insole to determine the user’s intention. ^3^ Novel force sensor that uses optical elements to amplify and measure small elastic deformations in the robot structure and that can be fully integrated as a structural element of the finger module. ^4^ PMA-Driven Exoskeleton powered by antagonistic PMAs: each PMA is connected to the structure basis through a force sensor to detect the real-time force information. An angular encoder is mounted along the joint axis to measure the angular position of the knee joint. ^5^ Vision-based control law for the precise control of a reaching motion in a ULE actuated by soft modules. ^6^ Load compensation and load information calculation for an ULE based on a six-axis force/torque sensor installed between the exoskeleton and the end-effector, which allows to calculate the weight of the load and the position of its centre of gravity relative to the exoskeleton and end-effector accurately.

**Table A6:** Main review publications on Exoskeleton theme.

Reference ID	Purpose *p*				Anatomical Districts *ad*														Device Type *dt*					Focus *f*			Design Solutions *ds*												
	1	2	3	4	1					2				3				4	1		2	3					1							2	3				4
						a	b	c	d		a	b	c		a	b	c			a	b			1	2	3		a	b	c	d	e	f			a	b	c	
Hussain_2021 [27]		x																							x														
Sun_2021a [219]		x								x	x	x							x	x					x		x						x		x		x		
Hu_2021 [81]	x				x		x												x	x					x		x	x							x	x			
Xiao_2021 [206]	x													x		x	x		x		x			x			x						x		x		x		
Liu_2021 [77]	x									x		x							x	x					x		x		x						x		x		
Wang_2021 [109]	x									x	x								x	x				x			x		x						x		x		
Guo_2021 [207]	x													x	x				x	x					x		x						x	x		x			
Li_2021a [51]	x													x		x			x		x			x			x						x		x		x		
Ferrero_2021a [177]	x	x								x	x	x	x						x		x			x			x					x			x			x	
Susanto_2021 [116]	x									x		x												x											x		x		
Li_2021b [178]	x									x		x							x						x										x		x		
Herbin_2021 [82]	x				x	x	x	x											x		x				x		x	x							x	x			
Zahedi_2021a [214]		x			x			x											x						x														
Zahedi_2021b [215]		x			x		x												x						x		x					x			x			x	
Coltelli_2021 [250]	x	x	x																						x														
Shao_2021 [220]		x								x			x												x		x						x						
Bin_2021 [106]	x		x		x		x							x		x			x		x			x	x									x					
Aguirre-Ollinger_2021 [69]		x								x		x							x						x		x	x							x		x		
Glowinski_2021 [248]										x	x	x	x						x							x													
Ferrero_2021b [179]	x	x								x	x	x	x						x		x			x			x					x			x			x	
Penzlin_2021 [79]		x								x	x	x							x						x		x				x				x		x		
Bützer_2021 [107]		x												x		x			x						x		x	x							x		x		
Gomez-Vargas_2021 [180]	x									x			x						x							x	x						x						
Sun_2021b [118]	x				x	x	x	x	x										x					x			x					x			x		x		
Asgher_2021 [236]		x												x		x			x					x			x	x					x		x		x		
Cao_2021 [94]	x									x	x	x							x	x					x		x						x		x		x		
Hunt_2021 [169]	x				x	x													x	x					x		x						x		x	x			
Staman_2021 [92]	x									x		x													x														
Dittli_2021 [237]		x												x		x			x	x					x		x	x											
Zhang_2021 [113]	x													x	x				x	x				x			x	x					x						
Qi_2021 [117]		x	x							x	x	x	x											x			x						x		x		x		
Zhao_2021c [238]			x		x		x																	x															
Li_2021c [181]	x									x	x	x	x						x	x				x															
Kawase_2021 [245]				x						x	x	x										x		x															
Williams_2021 [182]	x									x	x	x							x	x				x			x						x		x	x	x		
Khamar_2021 [183]	x									x	x	x							x	x					x		x					x		x	x		x		
Moggio_2021 [208]	x																									x													
Du_2021 [184]	x	x								x	x								x	x				x			x						x	x					
Chakarov_2021 [100]	x	x																	x	x	x				x														
Setiawan_2021 [170]		x												x		x			x	x					x		x	x						x	x		x		
Aftab_2021 [185]	x									x	x	x							x	x					x		x	x							x		x		
Tan_2021 [221]		x																	x		x				x		x	x							x			x	
Lee_2021 [186]	x	x								x			x						x	x					x		x	x											
Heo_2021 [187]	x									x		x							x		x				x		x					x			x		x		
Hamaya_2021 [111]	x	x			x	x	x												x		x			x			x	x											
Nunes_2020 [188]	x									x	x	x	x						x		x				x		x					x			x			x	
Choi_2020 [222]		x								x		x							x		x			x			x					x		x	x		x		
Zhao_2020 [97]	x									x		x							x		x				x		x	x							x		x		
Chen_2020a [171]	x				x	x	x												x	x					x		x	x						x	x		x		
Llorente-Vidrio_2020 [189]	x									x	x	x	x						x		x			x			x						x	x	x	x			
Park_2020 [172]	x				x	x	x	x	x										x		x	x			x		x					x			x	x			
Cao_2020 [96]	x									x	x	x							x	x					x		x	x					x		x		x		
Liu_2020 [68]		x			x	x	x												x		x				x		x			x					x		x		
Kim_2020 [243]				x	x		x												x		x				x		x					x			x			x	
Grazi_2020 [216]		x			x	x																x			x										x			x	
Orekhov_2020 [223]		x								x			x						x		x				x		x	x							x			x	
Yang_2020 [224]		x								x			x	x		x			x		x			x			x					x							
Ortiz_2020a [190]	x																		x		x			x										x					
Birouaş_2020 [209]	x													x		x			x		x				x		x	x					x						
Calanca_2020 [64]		x								x	x								x		x				x		x					x			x		x		
Ortiz_2020b [191]	x									x	x	x	x						x		x			x															
Barjuei_2020 [65]		x								x	x								x		x				x		x					x		x	x		x		
Yu_2020 [87]		x								x	x								x		x				x		x					x		x	x		x		
Chen_2020b [114]	x									x	x	x	x						x		x				x										x		x		
Yin_2020 [192]	x									x	x	x							x		x			x											x		x		
Xiang_2020 [225]		x								x	x	x							x		x				x		x					x			x		x		
DiNatali_2020 [226]		x								x	x	x	x						x		x				x		x	x							x		x	x	
Baser_2020 [70]	x	x								x	x	x	x						x		x				x		x		x					x	x		x		
Hyun_2020 [83]			x							x	x								x		x				x		x	x	x					x	x		x	x	
Wang_2020a [108]	x	x								x	x	x	x						x		x					x	x					x			x		x		
Asín-Prieto_2020 [193]	x									x			x						x		x					x									x		x		
Yahya_2020 [217]		x			x		x												x		x			x															
Xie_2020 [251]										x	x	x	x											x															
Gambon_2020 [194]	x									x	x	x							x		x			x											x		x		
Bhagat_2020 [173]	x				x		x												x		x			x			x						x		x		x		
Chen_2020c [99]	x				x	x	x												x	x					x		x	x											
Hsieh_2020 [228]		x								x	x								x		x				x		x	x		x					x			x	
Setiawan_2020 [210]	x													x		x			x	x					x		x	x							x				x
Li_2020 [195]	x									x	x	x							x		x				x		x					x			x		x		
Wang_2020b [241]			x							x	x	x							x		x					x									x	x			
Zhang_2020 [102]		x								x		x							x		x				x		x			x									
Lee_2020 [246]										x		x														x	x						x		x		x		
Pan_2020 [196]	x									x	x	x												x			x					x			x		x		
Chen_2019a [86]	x				x		x		x										x		x				x		x	x						x	x	x			
Hong_2019 [135]			x							x			x									x			x		x						x		x		x		
Al-Shuka_2019 [9]			x																						x														
Li_2019 [128]		x								x	x	x	x						x		x			x			x				x				x		x		
Moon_2019a [124]		x								x		x	x						x		x			x			x	x							x			x	
Crea_2019 [137]		x								x	x								x		x			x			x						x						
Zhang_2019 [75]		x								x	x								x		x				x		x					x			x		x		
Ismail_2019 [138]		x			x		x	x											x		x				x		x	x	x						x			x	
Lee_2019 [93]	x									x		x							x	x					x														
Pan_2019 [132]	x									x	x	x							x		x				x									x	x			x	
Moon_2019b [136]		x								x		x	x						x		x				x		x	x							x			x	
Rudd_2019 [139]	x													x		x			x	x					x		x	x											
Marconi_2019 [52]	x	x												x		x			x		x				x		x	x							x		x		
Liu_2019 [227]		x								x			x						x		x				x		x		x					x	x		x		
Wang_2019a [144]		x								x			x									x		x			x						x		x			x	
Wang_2019b [239]			x		x	x	x												x		x			x			x						x		x				
Khazoom_2019 [90]		x								x			x						x		x				x									x	x		x		
Al-Ayyad_2019 [151]		x								x		x							x		x				x		x					x			x		x		
Vantilt_2019 [72]		x								x	x	x	x						x		x				x		x		x	x				x	x	x			
Jarrett_2019 [115]	x			x	x		x												x		x			x			x	x							x		x		
Agarwal_2019 [1]	x													x		x			x		x					x	x	x					x	x					
Trigili_2019 [119]	x				x	x	x												x		x			x			x		x										
Nomura_2019 [229]		x								x	x	x	x						x		x			x											x		x		
Islam_2019 [154]		x			x	x	x																	x			x		x				x		x		x	x	
Chen_2019b [197]	x	x																	x		x				x		x				x								
Belogusev_2019a [66]	x	x																							x														
Belogusev_2019b [67]	x	x																	x		x				x														
Kazeminasab_2018 [110]	x	x																	x	x					x														
Muijzer-Witteveen_2018 [198]	x																		x		x			x															
Alamro_2018 [120]																										x								x	x				
Lemerle_2018 [55]	x													x	x				x						x		x	x					x		x			x	x
Hunt_2018 [140]		x			x	x		x		x		x	x						x	x					x								x	x	x				x
Manna_2018 [25]	x				x	x	x	x											x	x					x		x	x	x	x		x	x	x	x	x			
Long_2018a [240]			x							x	x	x	x						x		x			x									x	x	x			x	x
Ko_2018 [54]		x																x	x		x				x		x	x	x		x			x	x	x			
Long_2018b [89]		x								x	x	x	x						x		x				x	x	x			x		x		x	x				x
Yang_2018 [199]	x									x	x	x	x						x		x			x											x	x			
Copaci_2018 [218]		x			x		x												x		x			x		x	x	x											
Huang_2018 [230]		x								x		x							x		x				x	x							x		x			x	
Al-Fahaam_2018 [56]			x											x		x							x		x		x	x		x		x		x	x			x	x
Wang_2018a [34]										x	x	x							x							x													
Wang_2018b [35]										x	x	x							x							x													
Schrade_2018 [76]	x	x								x	x	x	x						x		x				x		x		x		x	x	x	x	x	x		x	
Choi_2018 [112]		x								x	x								x		x			x			x		x	x				x	x	x		x	
Lee_2018 [211]	x													x		x	x					x			x		x					x			x			x	
Díez_2018 [155]	x	x												x		x								x									x	x					
Toxiri_2018 [80]			x		x	x				x									x		x				x										x			x	
Yue_2018 [200]	x	x	x							x	x	x							x		x			x			x						x		x			x	
Zhu_2018 [249]										x														x															
Kuo_2018 [36]	x									x	x	x							x	x						x													
Jacobs_2018 [37]		x								x		x	x						x		x					x													
Kardan_2017 [231]		x								x		x	x						x		x				x	x	x		x						x			x	
Huang_2017 [201]	x	x								x		x	x						x		x				x		x						x	x					
Hsieh_2017 [73]	x				x	x													x						x		x						x		x	x			
Torrealba_2017 [202]	x									x		x							x	x					x		x		x										
Chen_2017a [71]	x									x	x	x	x						x						x		x		x						x	x		x	
Han_2017 [74]		x	x							x		x							x		x				x		x		x										
Oguntosin_2017 [103]	x	x			x				x										x		x				x		x						x		x			x	
Ahmed_2017 [242]			x							x	x	x							x		x			x		x									x			x	
Kim_2017 [84]	x		x	x						x	x	x							x						x	x									x			x	
Chen_2017b [38]										x		x							x							x									x			x	x
Hope_2017 [57]	x				x			x	x																x									x	x	x			x
Yang_2017 [146]			x							x	x	x	x						x		x				x														
Galle_2017 [247]										x			x						x		x					x													
Garate_2017 [39]	x	x								x	x	x	x						x		x					x								x					
Agarwal_2017 [49]	x													x	x				x	x					x		x	x							x			x	
Erdogan_2017 [203]	x									x			x						x		x					x	x	x											
Li_2017 [40]																			x							x													
Hunt_2017 [174]	x		x		x	x													x	x					x		x		x				x						
Zhu_2017 [88]			x							x		x							x		x				x									x					
Copaci_2017 [58]	x				x		x												x	x					x		x	x							x	x	x		x
Park_2017 [232]		x								x	x	x	x						x		x			x			x	x						x	x			x	
Khan_2016 [59]					x		x																	x			x				x				x				x
Lancini_2016 [204]	x									x	x	x	x						x		x			x											x			x	
Samadi_2016 [252]																										x													
Liu_2016 [153]	x	x																	x		x			x		x									x			x	
Keller_2016 [253]																										x													
Ben-Tzvi_2016 [157]	x			x										x		x			x					x			x	x					x		x				x
Nycz_2016 [160]	x													x		x			x	x					x		x	x						x					
Wang_2016 [50]	x													x	x											x	x	x					x		x			x	
Madani_2016 [41]	x									x		x							x							x													
Mineev_2016b [254]	x																							x															
Zhu_2016 [42]			x																x		x					x	x						x		x				x
Lu_2016 [91]										x	x	x	x												x	x													
Nordin_2016 [60]	x													x		x			x	x					x		x					x			x			x	x
Biryukova_2016 [212]	x													x		x										x									x				x
Agarwal_2015 [61]	x			x										x	x				x	x						x	x	x	x				x		x			x	x
Ben-Tzvi_2015 [53]				x										x		x								x			x	x					x	x	x			x	x
Jung_2015 [43]	x									x	x	x							x							x								x	x			x	
Kim_2015 [105]	x	x								x		x							x		x				x		x				x		x	x	x	x		x	
Aguilar-Sierra_2015 [101]	x		x							x	x	x	x												x		x	x			x			x					
Hwang_2015 [44]				x						x	x	x							x		x					x									x			x	
Susanto_2015 [45]				x										x		x			x							x													
Elnady_2015 [131]	x				x		x																	x			x		x					x	x			x	
Tamez-Duque_2015 [205]	x	x																	x		x			x											x			x	x
Villa-Parra_2015 [46]	x									x		x							x							x													
Mehmood_2015 [255]					x		x												x						x		x	x											
Cestari_2014 [78]		x								x		x							x					x	x		x		x										
Tung_2014 [233]		x								x	x	x														x	x	x			x		x	x	x			x	
Aubin_2014 [62]	x													x	x		x		x		x					x	x	x						x	x			x	x
Ertas_2014 [121]	x													x	x				x		x				x		x					x	x		x	x		x	x
Jones_2014 [133]	x													x	x				x	x						x	x	x							x	x			
